# Undetectable off-target effects induced by FokI catalytic domain in mouse embryos

**DOI:** 10.1186/s13059-024-03188-9

**Published:** 2024-02-20

**Authors:** Long Xie, Hu Feng, Zhifang Li, Di Li, Xiali Yang, Tanglong Yuan, Nana Yan, Chenfei He, Jitan Zheng, Zhenrui Zuo, Yaxuan Zheng, Yaqi Cao, Yangqing Lu, Xing Yao Xiong, Erwei Zuo

**Affiliations:** 1 Shenzhen Branch, Guangdong Laboratory for Lingnan Modern Agriculture, Key Laboratory of Synthetic Biology, Ministry of Agriculture and Rural Affairs, Agricultural Genomics Institute at Shenzhen, Chinese Academy of Agricultural Sciences, Shenzhen, China; 2 State Key Laboratory for Conservation and Utilization of Subtropical Agro-bioresources, Guangxi Key Laboratory of Animal Breeding, Disease Control and Prevention, College of Animal Science and Technology, Guangxi University, Nanning, 530004 China; 3 College of Animal Sciences and Technology, Huazhong Agricultural University, Wuhan, China

## Abstract

The FokI catalytic domain can be fused to various DNA binding architectures to improve the precision of genome editing tools. However, evaluation of off-target effects is essential for developing these tools. We use Genome-wide Off-target analysis by Two-cell embryo Injection (GOTI) to detect low-frequency off-target editing events in mouse embryos injected with FokI-based architectures. Specifically, we test FokI-heterodimers fused with TALENs, FokI homodimers fused with RYdCas9, or FokI catalytic domains alone resulting in no significant off-target effects. These FokI genome editing systems exhibit undetectable off-target effects in mouse embryos, supporting the further development of these systems for clinical applications.

**Supplementary Information:**

The online version contains supplementary material available at 10.1186/s13059-024-03188-9.

## Background

Wild-type FokI is a naturally occurring type IIS class endonuclease comprised of two distinct domains, including an N-terminal DNA-binding domain and a C-terminal domain that catalyzes nonspecific DNA-cleavage activity [1]. This FokI DNA-cleavage domain is essential for the effectiveness and broad application of ZFNs [2], as well as the later TALENs [3] and FokI-dCas9 (fCas9) [4] systems. Additionally, gene therapies that rely on this FokI activity are currently under evaluation in clinical trials, implying that FokI may hold still greater value in future human gene therapy applications.

Dimerization by the catalytic domain is required for FOKI endonuclease activity, and therefore the correct orientation and spacing between the dimerized FokI-based architectures is essential for the successful induction of double-strand breaks (DSBs). Initially, FokI homodimers were used to construct ZFNs for genome editing. However, the targeting specificity of FokI homodimers can be adversely affected by possible non-selective dimerization by monomers of the wild-type FokI cleavage domain [5]. In order to minimize or eliminate potential off-target cutting by homodimer-FokI-based architecture, a number of FokI variants have been developed, e.g., FokI^ELD^ and FokI^KKR^ [6], that only be functional when heterodimerized.

To further improve targeting selectivity and reduce off-target effects, heterodimer catalytic domains with increased pair-match requirements were developed and applied in ZFNs and TALENs. However, these heterodimer architectures also displayed substantial off-target genome editing [7]. It is likely that these off-target effects induced by FokI-based architectures may be related to mismatches between the DNA binding architecture (DBA) and an undesired locus. Alternatively, these effects may be caused by unexpected homodimerization among the FokI monomers in gene editing architectures (Additional file 1: Fig. S1). Dimerization between a FokI catalytic domain variant lacking its DNA binding domain and wild-type FokI monomers also results in higher rates of DNA cleavage than that of either homodime r[1], indicating that FokI can dimerize with other architectures even in the absence of its DNA binding region. Determining the source of these off-target effects and increasing DNA targeting specificity to minimize these effects is thus an essential step in the development of safe and efficient FokI-based architectures, especially for application in human gene therapies.

To address these issues, in the current study, we used Genome-wide Off-target analysis by Two-cell embryo Injection (GOTI), which provides high-sensitivity detection of off-target effects in the assessment of gene editing tools by editing one blastomere of two-cell mouse embryos. As the embryo develops, rare off-target sites in the edited blastomere are replicated in daughter cells, thus amplifying the off-target signal in sequencing analysis, such as reported in BE3 [8–10] and DdCBE [11] architectures. Here, we used GOTI to evaluate the off-target effects induced by heterodimeric FokI-based TALENs and homodimeric FokI-based RYdCas9 (fRYdCas9, derived from a nearly PAM-less RYdCas9 variant [12]), as well as for the FokI catalytic monomer. The findings in this study provide evidence valuable to risk evaluation of FokI-mediated gene editing relevant to both basic and clinical research applications.

## Results

To assess the off-target effects of FokI, we first examined its editing efficiency by fusing the catalytic domains of the FokI^ELD/KKR^ heterodimer to TALE arrays targeting the Rosa26 and MSTN locus in mice (Additional file 1: Fig. S2A, S2B). Briefly, plasmids containing the TALEN pairs were transfected into N2a cells. At 48h after plasmid delivery, EGFP+/mCherry+ cells were collected by fluorescence-assisted cell sorting (FACS; Additional file 1: Fig. S2C, D), and their genomic DNA was extracted. The gene editing effects were then evaluated by Sanger or next-generation sequencing (NGS) of the targeted loci. Sanger sequencing suggested robust gene cleavage in targeted loci (Additional file 1: Fig. S2C, D), while NGS analysis revealed that the cleavage sites in the Rosa26-TALENs and MSTN-TALENs loci were located between two TALE binding regions (Additional file 1: Fig. S3A, C) and showed a high frequency of Indels and nucleotide substitutions (Additional file 1: Fig. S3B, D). These results thus indicated that the FokI heterodimer-based TALENs architectures exhibited highly effective on-target gene editing.

To investigate the potential embryotoxicity of the FokI heterodimer-based TALE architectures, we next performed *in vitro* transcription (IVT) of TALENs targeting Rosa26 (Rosa26-ELD, Rosa26-KKR) or TALENs targeting MSTN (MSTN-ELD, MSTN-KKR). These mRNAs were injected with Cre into mouse zygotes as monomers alone or in pairs, with Cre alone serving as a control. At embryonic day 4.5 (E4.5), no significant differences were found in the percentage of blastocysts between the treated and untreated groups (Additional file 1: Fig. S4A). To evaluate the on-target effects of TALENs targeting Rosa26 and MSTN, injected embryos were picked and their DNA was extracted for nested PCR and Sanger sequencing, which showed that the Rosa26 and MSTN loci had indeed been cleavaged (Additional file 1: Fig. S4B). These results suggested that the FokI heterodimer fused with TALE architectures resulted in no significant embryotoxicity and displayed high on-target editing efficiency in embryos.

**Fig. 1:**
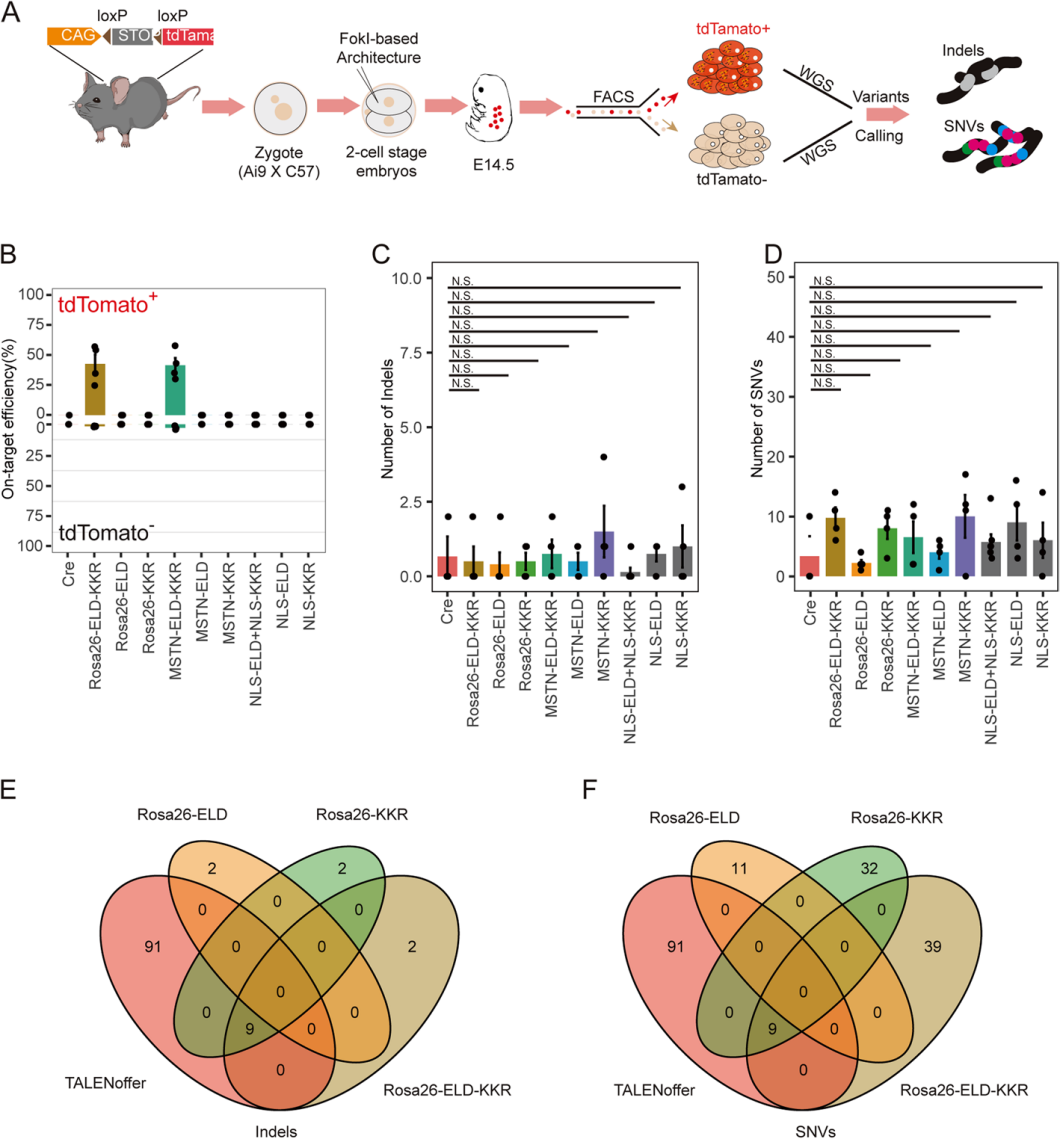
Undetectable off-target effect induced by heterodimer-FokI-based TALENs in mouse embryos. **A** Analyzed the DNA off-target effects of FokI-based genome editing tools using GOTI. **B** On-target efficiency of tdTomato+ and tdTomato− cell on the basis of WGS for the treated groups including Cre, Rosa26-ELD-KKR, Rosa26-ELD, Rosa26-KKR, MSTN-ELD-KKR, MSTN-ELD, MSTN-KKR, NLS-ELD-NLS-KKR, NLS-ELD, and NLS-KKR. **C, D**. Comparison of the total number of detected Indels (**C**) and SNVs (**D**) for the examined groups. **E**, **F** Overlap among Indels (**E**) and SNVs (**F**) of Rosa26-TALENs detected by GOTI under predicted off-targets by TALENoffer. All *P* values were calculated by two-sided Student’s *t*-tests. *n* ≥ 3 replicates were used in all experiments. Two Cre samples were derived from our previous study [8], while an additional Cre sample was newly derived in the present study

To explore the genome-wide off-targets of FokI heterodimer-based TALE architecture, we conducted GOTI assays by injecting mRNAs encoding the TALENs architecture, together with Cre mRNA, into one blastomere of two-cell embryos derived from Ai9 (CAG-LoxP-Stop-LoxP-tdTomato) mice (Fig. 1A). After injection, the presence of Cre protein will remove the “-Stop-” component, initiating the expression of tdTomato. Consequently, the detection of tdTomato+ cells will serve as a reliable indicator of successful the delivery of mRNA of genome editing tools and Cre (Additional file 1: Fig. S5A, S6A). The injected embryos were then transplanted into surrogate mice, while a subset of the injected embryos was left for tracking embryo development. After one blastomere injection, half the 8-cell embryos exhibited tdTomato expression in untransplanted embryos (Additional file 1: Fig. S5A, S6A). We then performed FACS to separate the tdTomato+ and tdTomato− progeny cells of the edited and non-edited blastomeres at E14.5. The on-target efficiency and off-target effects were examined by WGS (50× coverage) in all seven groups (Cre, Rosa26-ELD-KKR, Rosa26-ELD, Rosa26-KKR, MSTN-ELD-KKR, MSTN-ELD, MSTN-KKR; *n* ≥ 3 embryos per group) with subsequent indel/SNV calling in the tdTomato+ samples by three separate algorithms using tdTomato− samples from the same embryo as a reference (Additional file 1: Fig. S5D, S6D). Sanger sequencing was also used to validate the on-target efficiency in the tdTomato+ and tdTomato− cells (Additional file 1: Fig. S5C, S6C).

Sanger sequencing showed robust gene editing in Rosa26-ELD-KKR and MSTN-ELD-KKR groups, while the monomer architecture groups (Rosa26-ELD, Rosa26-KKR, MSTN-ELD, MSTN-KKR) present no DNA cleavage (Additional file 1: Fig. S3). The WGS results showed that on-target efficiency ranged from 30~50% in the Rosa26-ELD-KKR (42.53 ± 15.51%) and MSTN-ELD-KKR (41.4 ± 12.05%) groups (Fig. 1B), while no significant difference in off-target editing effects was observed among the treated groups (average number of Indels = 1; Fig. 1C). Furthermore, none of the Indels overlapped with the predicted off-target sites (Fig. 1E, Additional file 1: Fig. S7A), suggesting the possibility that the limited number of Indels detected in the Cre- and TALEN- treated samples may be the result of spontaneous mutation during genome replication in embryo development. Collectively, these results indicated that FokI-heterodimer-based TALE gene editing did not introduce any detectable Indels above background frequency in mouse embryos. Moreover, no significant differences in SNVs frequency were observed in groups treated with FokI-based architecture compared with the Cre group (Fig. 1D). On average, the Rosa26-ELD-KKR and MSTN-ELD-KKR harbored approximately 6–10 SNVs (Rosa-ELD-KKR, 9.75 ± 3.5; MSTN-ELD-KKR, 6.5 ± 5.26), which was comparable with that detected in the Cre group (3.33 ± 5.77). Additionally, none of the mutations were shared between any of the treated embryos, nor did they overlap with predicted off-target effects (Fig. 1F, Fig S7B). These results thus indicated that the FokI heterodimer-based TALENs architectures did not induce any detectable Indels or SNVs above the background.

In addition, the survival rate between NLS-ELD, NLS-KKR, NLS-ELD+NLS-KKR, and Cre at E4.5 (Additional file 1: Fig. S4A) showed no significant difference. In GOTI assays, WGS-based quantification of off-target editing effects identified 0~3 Indels per embryo in the FokI groups (NLS-ELD+NLS-KKR, 0.14 ± 0.38; NLS-ELD, 0.75 ± 0.50; NLS-KKR, 1 ± 1.41) (Fig. 1C), which was not significantly greater than that in the Cre-only group. Similarly, SNV calling identified an average of 6 SNVs in the NLS-ELD+NLS-KKR group (5.714 ± 3.35), whereas the NLS-ELD (9 ± 6.05) and NLS-KKR (6 ± 5.88) groups respectively had averages of 9 and 6 SNVs per embryo, none of which were significantly different from the Cre-only control group (Fig. 1D).

**Fig. 2:**
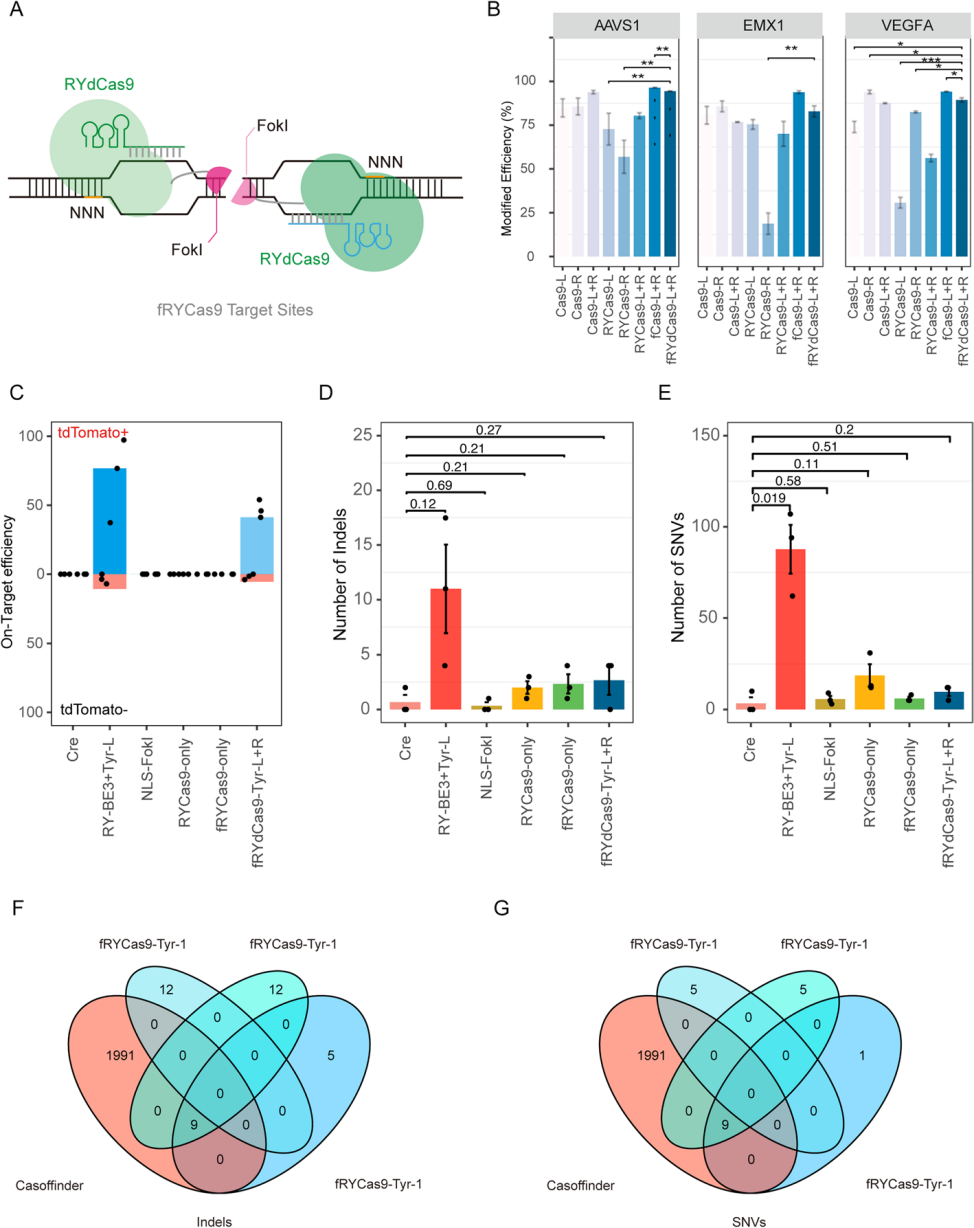
Undetectable off-target effect induced by homodimer-FokI-based fRYdCas9 in mouse embryos. **A** Schematic of homodimer fRYdCas9. **B** On-target efficiency of Cas9, RYCas9, fCas9, and fRYdCas9 for genes including AAVS1, EMX1, and VEGFA in HEK293T cells. **C** On-target efficiency of tdTomato+ and tdTomato− cell for the treated groups including Cre, Fokl-Homo, RYCas9, fRYdCas9, and fRYdCas9-Tyr in mouse embryos. **D**, **E** Comparison of the total number of detected Indels (**D**) and SNVs (**E**) for all groups. **F**, **G** Overlap among Indels (**F**) and SNVs (**G**) of fRYdCas9-Tyr-L+R detected by GOTI with predicted off-targets by Casoffinder. All *P* values were calculated by two-sided Student’s *t*-tests. *n* ≥ 3 replicates were used in all experiments.Two Cre samples were derived from Zuo et al. [8]

Other studies have proposed that the easier dimerization of the FokI-homodimer-based architecture could potentially result in a higher frequency of off-target effects than FokI heterodimer-based architectures [5, 6]. However, another recent report described a high-specificity gene editing tool that relies on a FokI-homodimer-based CRISPR/dCas9 (fCas9) [4, 13], although its application has been limited by strict requirements for two PAMs and a 10–40-nt spacer. To overcome the constraint imposed by target site recognition of a protospacer-adjacent motif (PAM), we used a nearly PAM-less (NNN, NRN > NYN) dead RYCas9 [12] (RYdCas9) in place of dCas9 to construct FokI-RYdCas9 (fRYdCas9) (Fig. 2A). This fRYdCas9 architecture was then used to target AAVS1 (93.978 ± 0.749%), EMX1 (82.872 ± 5.486%), and VEGFA (88.149 ± 0.429%) in HEK293T cells, which resulted in on-target editing efficiency comparable to that of Cas9 (AAVS1-L, 88.795 ± 8.584%; AAVS1-R, 85.659 ± 8.342%; EMX1-L, 80.515 ± 2.212%; EMX1-R, 85.736 ± 5.443%; VEGFA-L, 73.979 ± 5.578%; VEGFA-R: 93.931 ± 1.815%), RYCas9(AAVS1-L, 72.730 ± 15.714%; AAVS1-R, 56.906 ± 16.384%; EMX1-L, 75.438 ± 4.869%; EMX1-R, 18.761 ± 10.625%; VEGFA-L, 30.737 ± 5.279%; VEGFA-R: 82.486 ± 0.943%), or fCas9 (AAVS1, 96.468 ± 0.162%; EMX1, 93.853 ± 1.323%; VEGFA, 94.172 ± 0.315%) at target loci (Fig. 2B). It is interesting to note that the editing activity of RYCas9+sgRNA-L and RYCas9+sgRNA-R was relatively low, while fRYdCas9+sgRNA-L+sgRNA-R exhibited higher activity in target sites hAAVS1, hEMX1, and hVEGFA. For SpCas9 and RYCas9, employing a double sgRNA strategy may enhance the likelihood of editing the target locus, resulting in a higher percentage of modifications observed in the gene locus AAVS1 (SpCas9:93.867 ± 1.743%; RYCas9:80.425 ± 2.8714%), EMX1 (SpCas9:76.759 ± 0.409%; RYCas9:70.037 ± 12.376%), and VEGFA (SpCas9:87.571 ± 0.455%; RYCas9:56.202 ± 3.722%) (Fig. 2B). However, gene editing with fRYdCas9 only occurs when both sgRNAs bind to the target locus. This suggests that the formation of FokI dimer possibly increased the DNA binding affinity of fRYdCas9 and presented higher gene editing efficiency.

For mouse embryos, examination of on-target effects in cells treated with FokI homodimer fusion protein by injecting fRYdCas9 mRNA with a sgRNA pair targeting the Tyr gene (Tyr-L+R) in mouse zygotes showed that blastocyst rates were not significantly different from that in the untreated control group (Additional file 1: Fig. S9A). Additionally, Sanger sequencing of E4.5 embryos in the fRYdCas9 group showed high efficiency of on-target editing (Additional file 1: Fig. S9B).

To further investigate potential off-targets effects of homodimer FokI in the genome, we used GOTI to assess the FokI, RYCas9-BE3+Tyr-L, RYCas9-only, fRYdCas9-only, and fRYdCas9-Tyr-L+R groups. For each group, coding mRNAs and sgRNAs were co-injected into one blastomere of Ai9 mouse embryos at the two-cell stage. At the 8-cell stage, the tdTomato+ and tdTomato− blastomeres were evenly distributed in the injected embryos (Additional file 1: Fig. S10A). At E14.5, tdTomato+ and tdTomato− cells from transplanted GOTI embryos were sorted by FACS (Additional file 1: Fig. S10B) and sequenced by WGS (Additional file 1: Fig. S10B). Preliminary Sanger sequencing of PCR products of the Tyr locus targeted by fRYdCas9-Tyr-L+R showed efficient on-target editing (Additional file 1: Fig. S10C).

In WGS data, on-target editing efficiency reached 47% (47.03 ± 6.47%) in tdTomato+ cells in the fRYdCas9-Tyr-L+R group, while no editing was detected in tdTomato− cells. Evaluation of off-target editing showed no significant difference in Indels or SNVs counts which were found in any treatment group compared to that in the Cre-only controls (Fig. 2D, E). Under fRYdCas9-Tyr-L+R administration, 0–4 Indels (2.67 ± 2.31) and 5-12 SNVs (9.67 ± 4.04) per embryo were identified. In the no sgRNA group, injection with fRYdCas9 mRNA alone also generated no significant difference in off-target effects. Finally, all of the mutations of Indels and SNVs detected in GOTI assays were compared with the top 2000 off-target sites predicted by Casoffinder (based on left- and right-sgRNA sequence) and found no overlaps between the predicted and observed loci (Fig. 2F, G). These results suggested that FokI homodimer-based RYdCas9 architecture could provide high on-target editing efficiency with no detectable off-target effects beyond the background variants in mouse embryos.

## Discussion

FokI, as either a homodimer or a heterodimer (in more recent constructs), has been used to produce genome editing applications targeting human pathogens. FokI-based architectures, such as TALENs, continue to show potential for clinical translation in gene therapies due to their high-efficiency on-target editing and their small size that is conducive for delivery in humans. Previous reports have shown that the ZFNs/TALENs/fCas9 systems result in varying degrees of off-target effects. Whether these off-targets are induced by unexpected DNA binding activity by DBA domains or through undesirable FokI dimerization remains unclear. However, the unpredictable dimerization of FokI that potentially leads to random off-target effects reinforces concerns about its clinical application.

In this study, the highly sensitive and unbiased GOTI method revealed that off-target genome editing by FokI architectures is limited in mouse embryos, with neither FokI monomers nor FokI homodimer-/heterodimer-DBAs (TALEN/fRYdCas9) introducing any detectable increase in Indels or SNVs. These results suggest that FokI monomers rarely spontaneously dimerize to cleave DNA in genome editing of mouse embryos, supporting the likelihood that off-target effects of FokI-DBAs may be the result of unpredicted DNA binding by the DBA.

Following the hypothesis that FokI monomers more readily homodimerize and consequently increase cleavage at undesired DNA loci, considerable research attention has focused on optimizing FokI heterodimers to decrease the frequency and likelihood of off-target editing events. However, our results indicate that FokI monomers in fact rarely spontaneously homodimerize, and these homodimers do not introduce more off-target effects than heterodimers, suggesting DBA design may play a larger role than previously recognized in off-target effects, and improving DBA design is necessary to minimize off-target editing. Thus, more advanced design methods, such as deep-learning or structural re-design, may facilitate improvements to DBA design that potentially eliminate off-target effects altogether in FokI-based genome editing.

## Conclusions

The study employed the Genome-wide Off-target analysis by Two-cell embryo Injection (GOTI) method to meticulously examine potential off-target editing events in mouse embryos treated with various FokI-based architectures. Remarkably, the results demonstrated that architectures such as FokI-heterodimers fused with TALENs, FokI homodimers fused with RYdCas9, and even FokI catalytic domains alone exhibited no discernible off-target effects of significance. These compelling findings not only underscore the substantial potential of these FokI genome editing systems for clinical applications but also encourage their further advancement in research and development.

## Methods

### Plasmid construction

The TALEN plasmid targeting mice Rosa26 gene was constructed based on the TALEN-mROSA26-ELD and TALEN-mROSA26-KKR (gifts from Radislav Sedláček, Addgene #60025/#60026). CMV-mCherry and CMV-EGFP genome components were inserted into TALEN-mROSA26-ELD and TALEN-mROSA26-KKR, respectively. The TALE regions targeting MSTN were subcloned from pEGFP-2A-TAL6-MSTN (a gift from Renzhi Han, addgene #72597) and constructed into plasmid backbone including CMV-mCherry and CMV-EGFP genome component, respectively. The RYCas9 and fRYdCas9 were constructed based on the PX330 plasmid (PX330 was a gift from Feng Zhang; Addgene plasmid #42230)) according to the mutations reported in the literatur e[12]. Briefly, to construct fRYdCas9, FokI was fused to the N-terminus of RYdCas9 via a 32-amino acid linker sequence. Subsequently, a mCherry fluorescent protein was fused to the C-terminus of RYdCas9 using a T2A linker. For sgRNA plasmid construction, sgRNA oligos were annealed and inserted before scaffold (clone from px330) by T4 ligations. All these plasmids were extracted following the manuals of E.Z.N.A Endo-free plasmid mini kit.

### Cell culture, transfection, and genotyping

N2a or HEK293T cells were cultured in DMEM (high glucose) medium supplied with 10% fetal bovine serum (FBS). For N2a cell transfection, a total of 2 μg plasmids (TALEN-ELD:TALEN-KKR = 1:1) were transfected into N2A cells per well in 12-well plates. In HEK293T transfection, for per wells in 12-well plates, 2 μg Cas9 or RYCas9 plasmid was co-transfected into cells with 1 μg of sgRNA, fCas9 or fRYdCas9 plasmid was transfected into cells with 0.5 μg of Tyr-L-sgRNA and 0.5 μg of Tyr-R-sgRNA. Forty-eight hours after transfection, positive cells were isolated via FACS, and the DNA of cells was extracted according to the manufacture of DNeasy blood and tissue kit (catalog number 69504, Qiagen). The genotypes of DNA from transfected cells were determined by Nested PCR. In the first round of PCR amplification, Extaq (Takara) was activated at 95 °C for 3 min, then performed PCR for 30 cycles, at 95 °C for 30 s, 55°C for 30 s, 72 °C for 1 min, and a final extension at 72 °C for 5 min after cycles. The second round of PCR was performed using the same program with an inner nested primer. The PCR products were purified for Sanger Sequencing genotyping. Next-generation sequencing data were analyzed using the CRISPResso 2[14] (http://crispresso.pinellolab.org/submission).

### GOTI experimental design

All the experimental groups were treated using the same procedure for the GOTI examination. Briefly, the mixture of mRNA was injected into one blastomere of a 2-cell embryo, which was derived from Ai9 male mice mating with wild-type female mice. 2~4 h after injection, the injected embryos were transplanted into C57 surrogacy mice. In E14.5, cells from transplanted embryos were collected for FACS to isolate the tdTomato+ and tdTomato− cells, respectively. The off-target Indels and SNVs were identified by comparing the tdTomato+ cells with tdTomato− cells using three variant calling algorithms as indicated (Mutect2, Scalpel, and Strelka for Indels, and Mutect2, Lofreq, and Strelka for SNVs). In addition, the sufficient sequencing coverage at the off-target sites was re-confirmed by using Integrative Genomics Viewer (IGV).

### Animal care

Female C57BL/6 mice (4 weeks old) and homozygous Ai9(B6.Cg-Gt(ROSA)26Sortm9 (CAG-td-Tomato) Hze/J; JAX strain 007909) male mice were used for embryo collection. ICR females were used as recipients. The use and care of animals complied with the guidelines of the Biomedical Research Ethics Committee of Agricultural Genomics Institute in Shenzhen, Chinese Academy of Agricultural Sciences.

### Generation of mRNA and sgRNA

For all TALEN groups (Rosa26-ELD, Rosa26-KKR, MSTN-ELD, MSTN-KKR), FokI groups (NLS-ELD, NLS-KKR, NLS-FokI), RY-Cas9 and fRYdCas9, plasmids including a T7 promoter and corresponding architecture were respectively linearized as temples for IVT processes. For Cre, the T7 promoter was added to the N-terminal of the Cre coding region by PCR amplification, and PCR products were purified and used as temples for IVT. MESSAGE mMACHINE T7 ULTRA kit (Life Technologies) was used for the above architecture mRNA generation. For the IVT of sgRNA, The T7 promoter was added to the sgRNA template by PCR amplification of px330 (a gift from Feng Zhang; Addgene plasmid #42230), using primers listed as follows. The T7-sgRNA PCR product was purified and used as the temple of IVT using the MEGA shortscript T7 kit (Life Technologies). All IVT products of mRNA and SgRNA were purified using the MEGA clear kit (Life Technologies) and eluted in RNase-free water.

**Table:** 

Name	Sequence (5′-3′)
Cre IVT F	TAATACGACTCACTATAGGGAGACAGATCACCTTTCCTATCAACC
Cre IVT R	TCGGTATTTCCAGCACACTGGA
Tyr-L IVT F	TAATACGACTCACTATAGGGGCATCTCTCCAATCCCAGTAGTTTTAGAGCTAGAAATAG
Tyr-R IVT F	TAATACGACTCACTATAGGGtgCACAGATGAGTACTTGGGGTTTTAGAGCTAGAAATAG
sgRNA IVT R	TCTAGCTCTAAAACAAAAAAGCACC

### Two-cell embryo injection, embryo culturing, and embryo transplantation

For embryo collection, C57BL/6 females were preliminarily superovulated and mated with homozygous Ai9 males, 24 h after hCG injection, and fertilized embryos were collected from oviducts. For 2-cell embryo injection, all TALEN groups (Rosa26-ELD, Rosa26-KKR, MSTN-ELD, MSTN-KKR, or their combinations), FokI groups (NLS-ELD, NLS-KKR, NLS-FokI, or their combinations), RYCas9-only and fRYdCas9-only groups were settled concentration as 100 ng/μL and co-injected with the mRNA of Cre (2 ng/μL) into one blastomere of 2-cell embryo 48 h post-hCG injection. In the fRYdCas9 group, fRYdCas9 (100 ng/μL), Tyr-L sgRNA (100 ng/μL), and Tyr-R sgRNA (100 ng/μL) were co-injected with Cre (2 ng/μL). During the injection, embryos were transferred into a droplet of M2 medium containing 5 μg/mL cytochalasin B (CB), and the FemtoJet microinjector (Eppendorf) was turned into constant flow settings. The injected embryos were cultured in KSOM medium with the amino acid at 37 °C under 5% CO_2_ for 2 h and then transferred into oviducts of pseudo-pregnant ICR females at 0.5 dpc.

### Embryo genotyping

For embryo genotyping, in E4.5, a single blastocyst was transferred into 4 μl lysis buffer (0.1% Triton X-100, 0.1% TWEEN20, 4 μg/mL proteinase K), and perform the following program to expose DNA: 55 °C 30 min, 95 °C 10 min, 4 °C ∞. The nested PCR of targeting loci was performed for embryo cell genotyping. In the first round of PCR amplification, Extaq (Takara) was activated at 95 °C for 3 min, then performed PCR for 30 cycles, at 95 °C for 30 s, 55 °C for 30 s, 72 °C for 1min, and a final extension at 72 °C for 5 min after cycles. The second round of PCR was performed using the same program with an inner nested primer. The PCR products were purified for Sanger Sequencing genotyping.

### Fluorescence-activated cell sorting (FACS)

In embryo day 14.5 (E14.5), tissues from the embryo were dissociated into small pieces and digested in 5 mL Trypsin-EDTA (0.05%) at 37 °C for 30 min. The digestion was stopped by adding 5 mL of DMEM medium supplied with 10% Fetal Bovine Serum (FBS). Fetal tissues were then homogenized by pipetting 30–40 times through a 1mL pipette tip, 1000g centrifuged for 6 min, discarded supernatant, and re-suspended in DMEM supplied with 10% FBS. Next, after filtering through a 40-μm cell strainer, the tdTomato+/tdTomato− cells were isolated by FACS.

### Whole genome sequencing (WGS) and data analysis

The genomic DNA of tdTomato+/tdTomato− cells was extracted by using the DNeasy blood and tissue kit (catalog number 69504, Qiagen) according to the manufacturer's instructions. WGS was performed at mean coverages of 50× by BGI DNBSEQ-T7. The qualified sequencing reads in WGS data were mapped to the reference genome (GRCm39) by using BWA (v0.7.17), and Picard tools (v2.25.7) were then used to sort and mark duplicates of the mapped BAM files. To save computing time and resources, Strelka (v2.9.10) was first run for the detection of whole genome de novo Indels and SNVs. Then 200 bp upstream and downstream of the mutation location were selected as candidate regions. To call variants with high confidence, Mutect2 (v4.2) and Lofreq (v2.1.5) were run for the detection of SNVs with candidate regions. In parallel, Mutect2 and Scalpel (v0.5.4) were run for the detection of Indels with candidate regions. The variants that overlap in the three algorithms would be considered true SNVs or Indels. In the variants calling, we used tdTomato− data as a control to identify the mutations that appeared in tdTomato+ data for each pair of samples. Furthermore, all identified and overlapped SNVs and Indels were confirmed by manual realignment, and variants locus in repeat sequence array would be removed.

To predict the off-target sites, TALENoffe r[15] (http://www.jstacs.de/index.php/TALENoffer) was applied in Rosa26 and MSTN TALEN group, and Casoffinder was used in the fRYdCas9-Tyr-L+R group. For TALEN groups, the top 100 off-target sites overlapped with the Indels or SNVs detected in GOTI examinations. For the fRYdCas9 group, the top 2000 off-target sites predicted by Casoffinde r[16] (http://www.rgenome.net/cas-offinder/) based on the Tyr-L-sgRNA and Tyr-R-sgRNA were collected to overlap with the Indels or SNVs detected in GOTI examinations.

### Statistical analysis

R version 4.2.1 (https://www.r-project.org) was used to conduct all the statistical analyses in this study. All tests conducted were two-sided, and the significant difference was considered at *P* < 0.05.

### Supplementary information

**Additional file 1.** Supplementary figures S1-S11 and tables S1-S4.

**Additional file 2.** Details of sequencing data.

**Additional file 3.** Review history.

## Data Availability

All data generated or analyzed during this study are included in this published article (and its additional information files). CRISPResso2 is an open-source software pipeline designed to enable rapid and intuitive interpretation of genome editing experiments and is available on the GitHub website (https://github.com/pinellolab/CRISPResso2). BWA is a software package for mapping DNA sequences against a large reference genome and is available on GitHub (https://github.com/lh3/bwa). Strelka, Mutect2, and Lofreq are SNV calling packages with different algorithms, all of which are available on GitHub at https://github.com/Illumina/strelka, https://github.com/NCI-GDC/mutect2-cwl, and https://github.com/CSB5/lofreq, respectively. Scalpel is a software package for detecting INDELs (insertions and deletions) and is available at https://scalpel.sourceforge.net/. Casoffinder is a tool that searches for potential off-target sites of Cas9 RNA-guided endonucleases (RGENs) from different species and genomes. We used the web-version of this tool available at http://www.rgenome.net/cas-offinder. TALENoffer is a tool for genome-wide prediction of TAL effector nuclease (TALEN) off-target sites, and it can be accessed at http://www.jstacs.de/index.php/TALENoffer. The raw WGS fastq files used for the analysis have been deposited with the National Center for Biotechnology Information (NCBI) under the accession number PRJNA893392 [17]. Detailed information about the raw data is listed in Additional file 2.
