# Supplementary material for: Undetectable off-target effects induced by FokI catalytic domain in mouse embryos
Source: Genome Biol. 2024 Feb 20;25:51. doi: 10.1186/s13059-024-03188-9 (PMC10877887; doi:10.1186/s13059-024-03188-9)
Supplement: Supplementary file 1 — Additional file 1. Supplementary figures S1-S11 and tables S1-S4. [file 13059_2024_3188_MOESM1_ESM.docx]

**Supplementary Information**


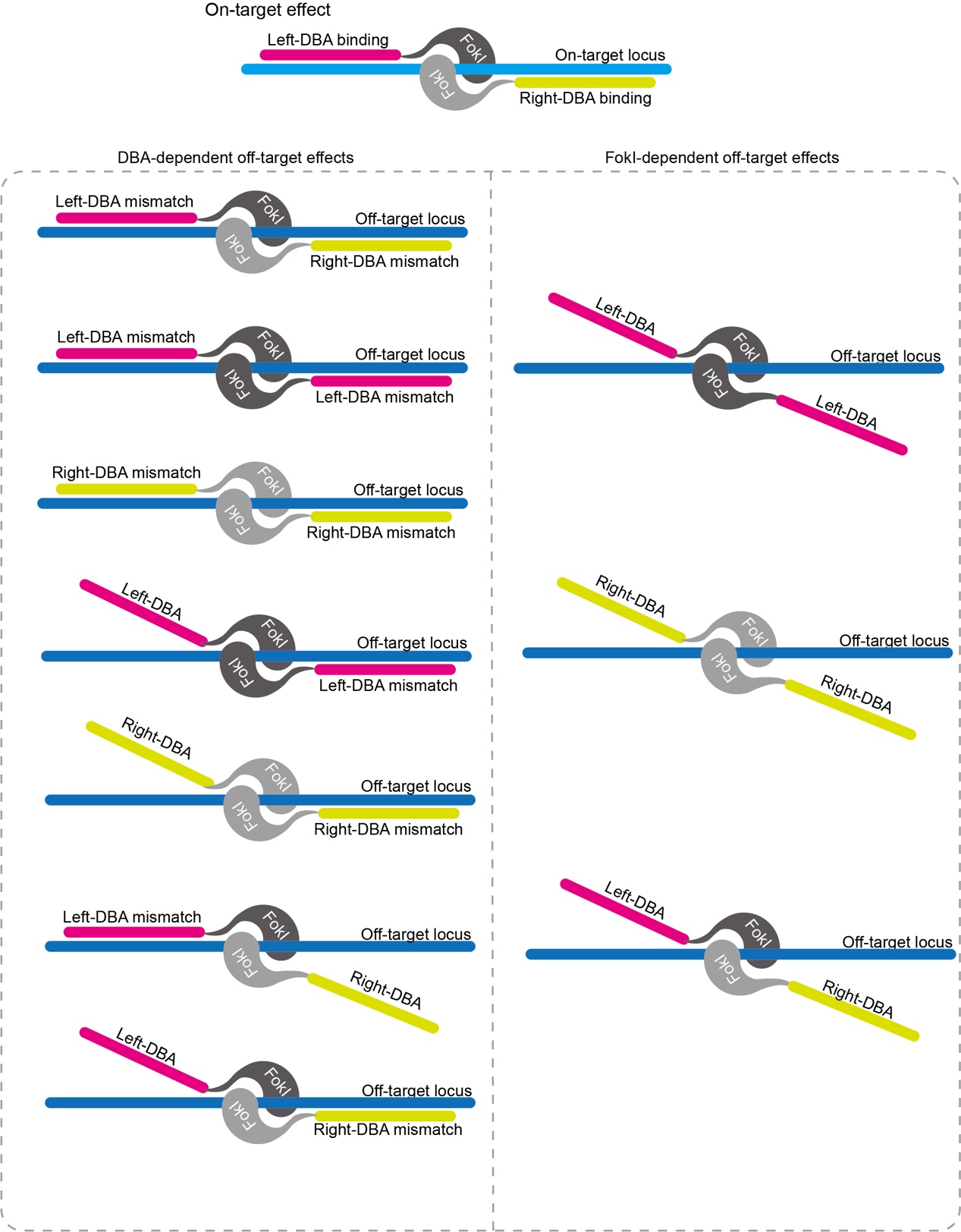


**Fig S1.** The on-target and potential off-target modules of heterodimer and homodimer-FokI-based genome editing architectures.

**
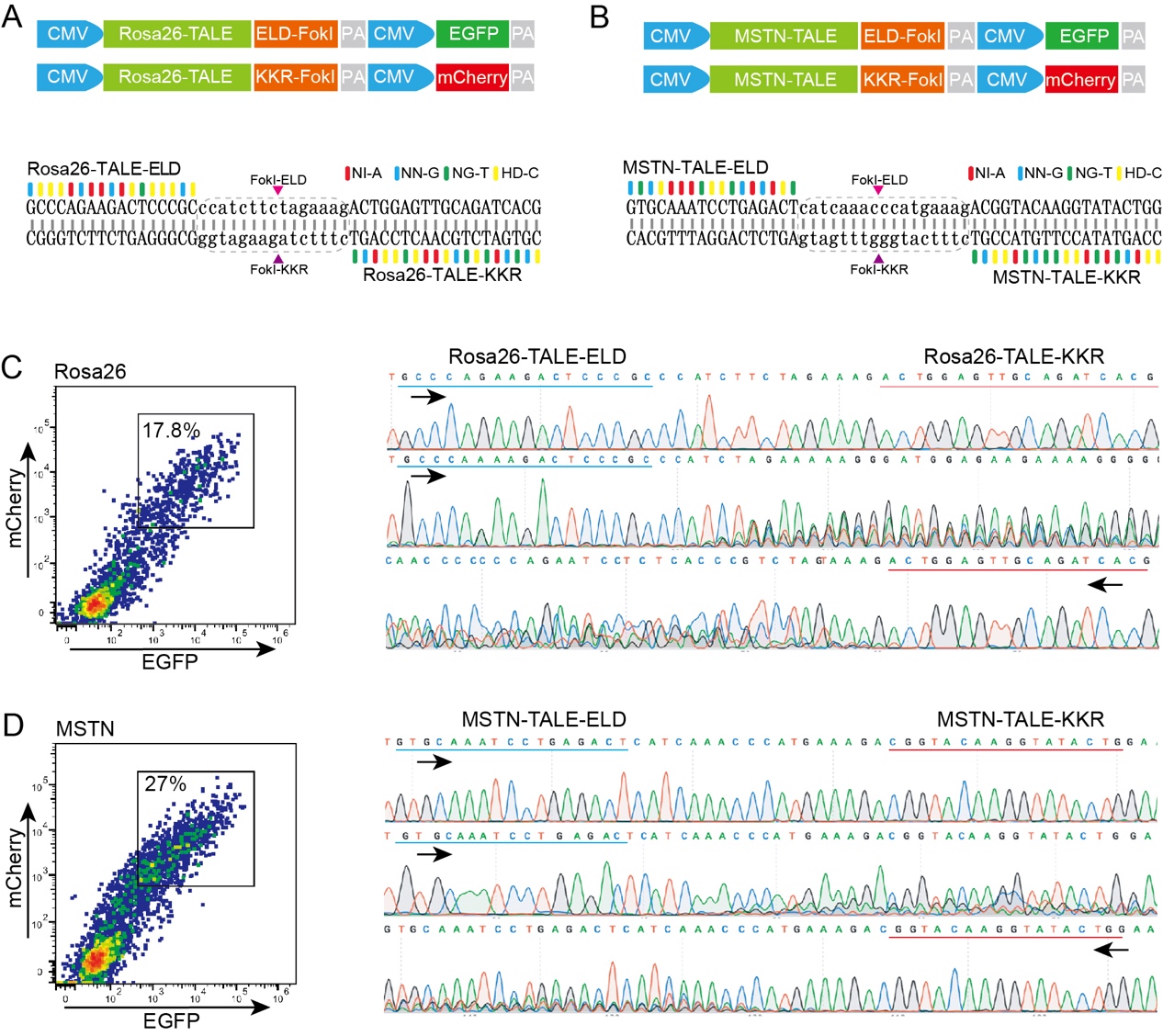
**

**Fig S2. Heterodimer-FokI-based TALENs architectures mediate genome editing in N2a cells.**

**A.** Schematic showing the architecture of Rosa26-ELD and Rosa26-KKR. **B.** Schematic presenting the architecture of MSTN-ELD and MSTN-KKR. **C.** FACS analysis of N2a cells transfected by Rosa26-ELD-KKR (left) and Sanger sequencing showing its editing effect (right). (arrow represents the Sanger sequencing direction). **D.** FACS analysis of N2a cells transfected by MSTN-ELD-KKR (left) and Sanger sequencing showing its editing effect (right). (arrow represents the Sanger sequencing direction)


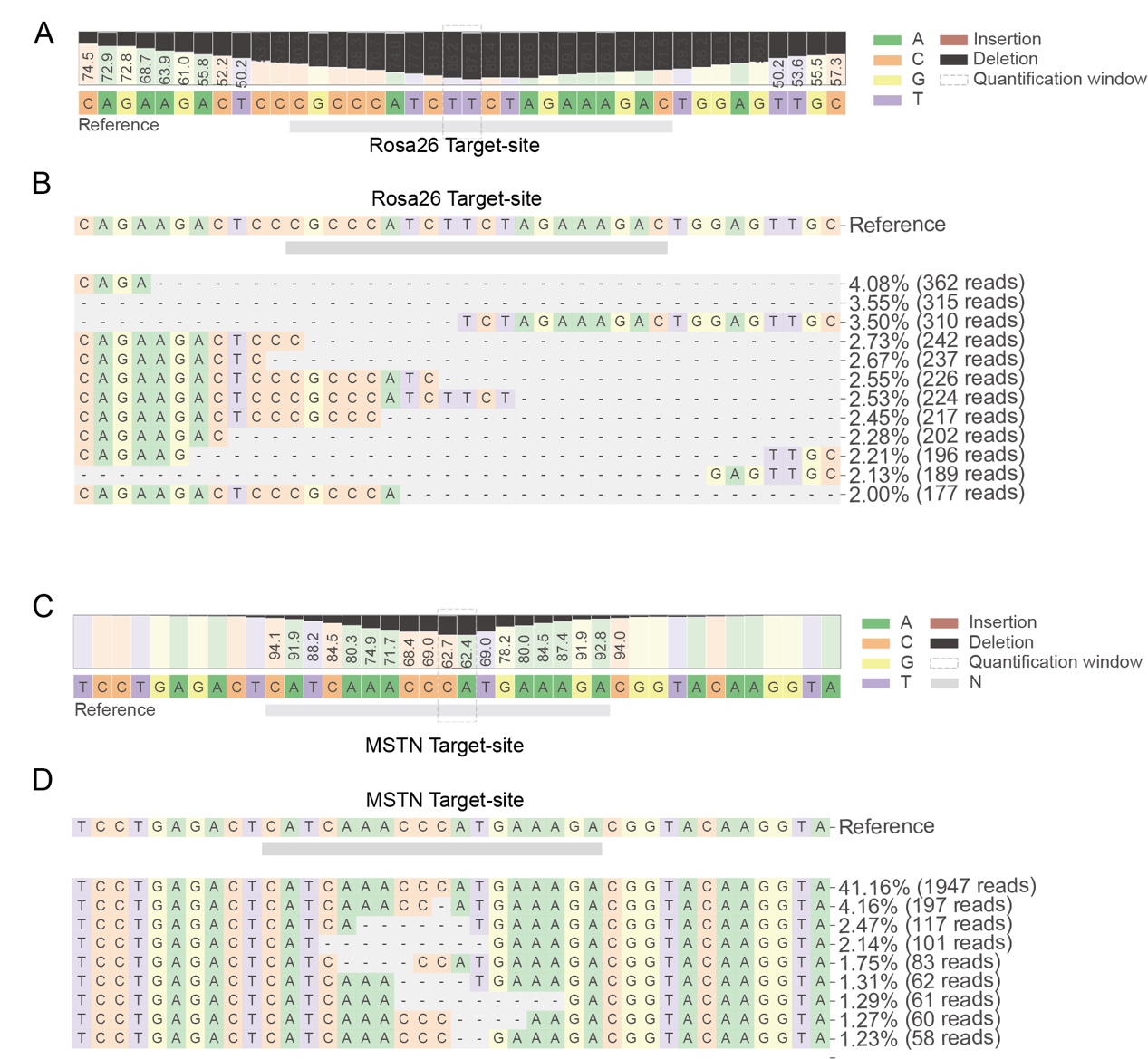


**Fig S3. The on-target analysis of Heterodimer-FokI-based TALENs architectures in N2a cells.**

**A.** Nucleotide frequency in quantification window of Rosa26-ELD-KKR in N2a cells. **B.** NGS analysis showing editing effects of Rosa26-ELD-KKR in N2a cells. **C.** Nucleotide frequency in quantification window of MSTN-ELD-KKR in N2a cells. **D.** NGS analysis showing editing effects of targeted MSTN-ELD-KKR in N2a cells.


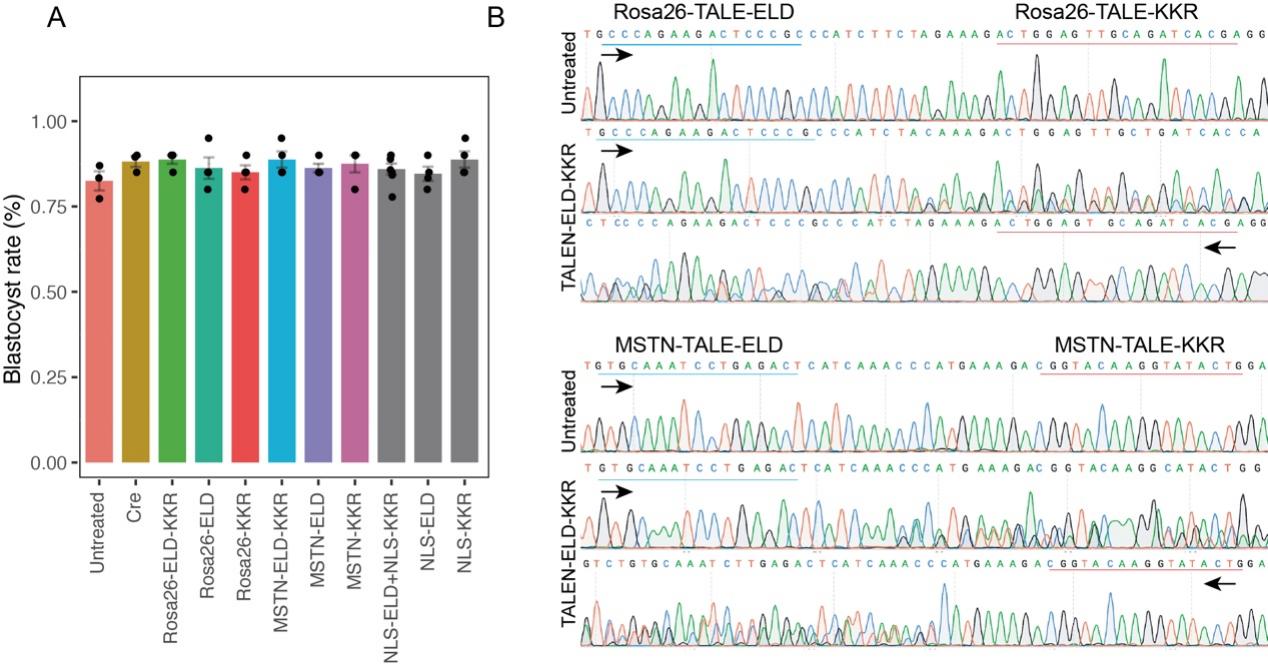


**Fig S4. Heterodimer-FokI-based TALENs architectures mediated genome editing in mouse zygotes.**

**A.** Blastocyst rate of treated by Cre, Rosa26-ELD-KKR, Rosa26-ELD, Rosa26-KKR, MSTN-ELD-KKR, MSTN-ELD, MSTN-KKR, NLS-ELD+NLS-KKR, NLS-ELD, NLS-KKR, and untreated groups. **B.** Sanger sequencing analysis showing the gene editing effects of Rosa26-ELD-KKR and MSTN-ELD-KKR in E4.5 embryos (arrow represented Sanger sequencing direction). All *P* values were calculated by two-sided Student’s *t*-tests. n ≥ 3 replicates were used in all experiments.

**
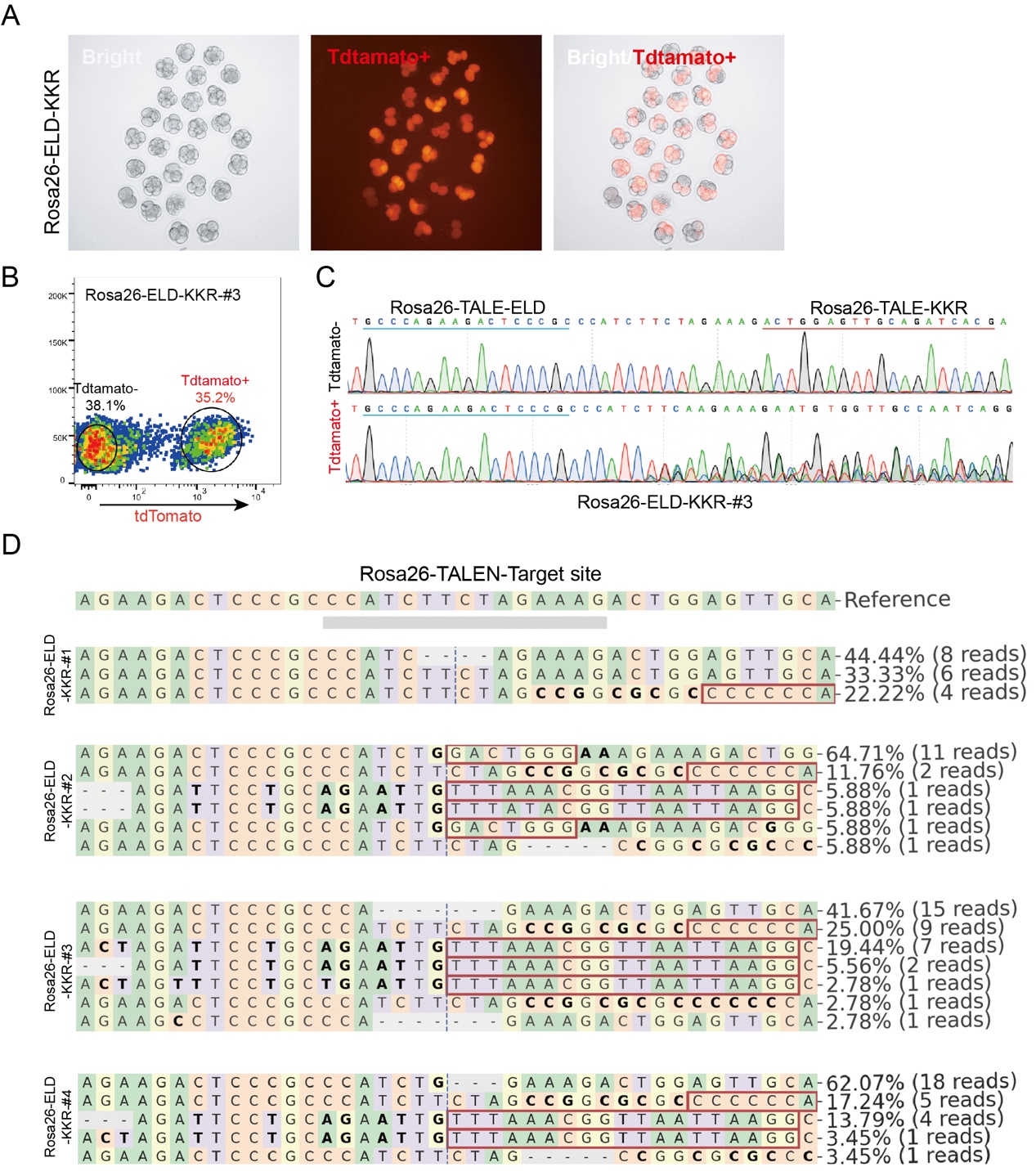
**

**Fig S5. Heterodimer-FokI-based TALENs architectures mediated Rosa26 gene editing in one blastmere of 2-cell mouse embryos.**

**A.** The tdTamato expression of the 8-cells embryos which were edited one blastomere by Rosa26-ELD-KKR + Cre at the two-cell stage observed by fluorescent microscope. **B.** FACS analysis of E14.5 embryos treated by Rosa26-ELD-KKR + Cre. **C.** Sanger sequencing showed the on-target effects of tdTomato^+^ and tdTomato^-^ embryo cells treated by Rosa26-TALE-ELD-KKR. **D.**WGS analysis showing editing effect of targeted Rosa26-ELD-KKR in tdToamato+ embryo cells.

**
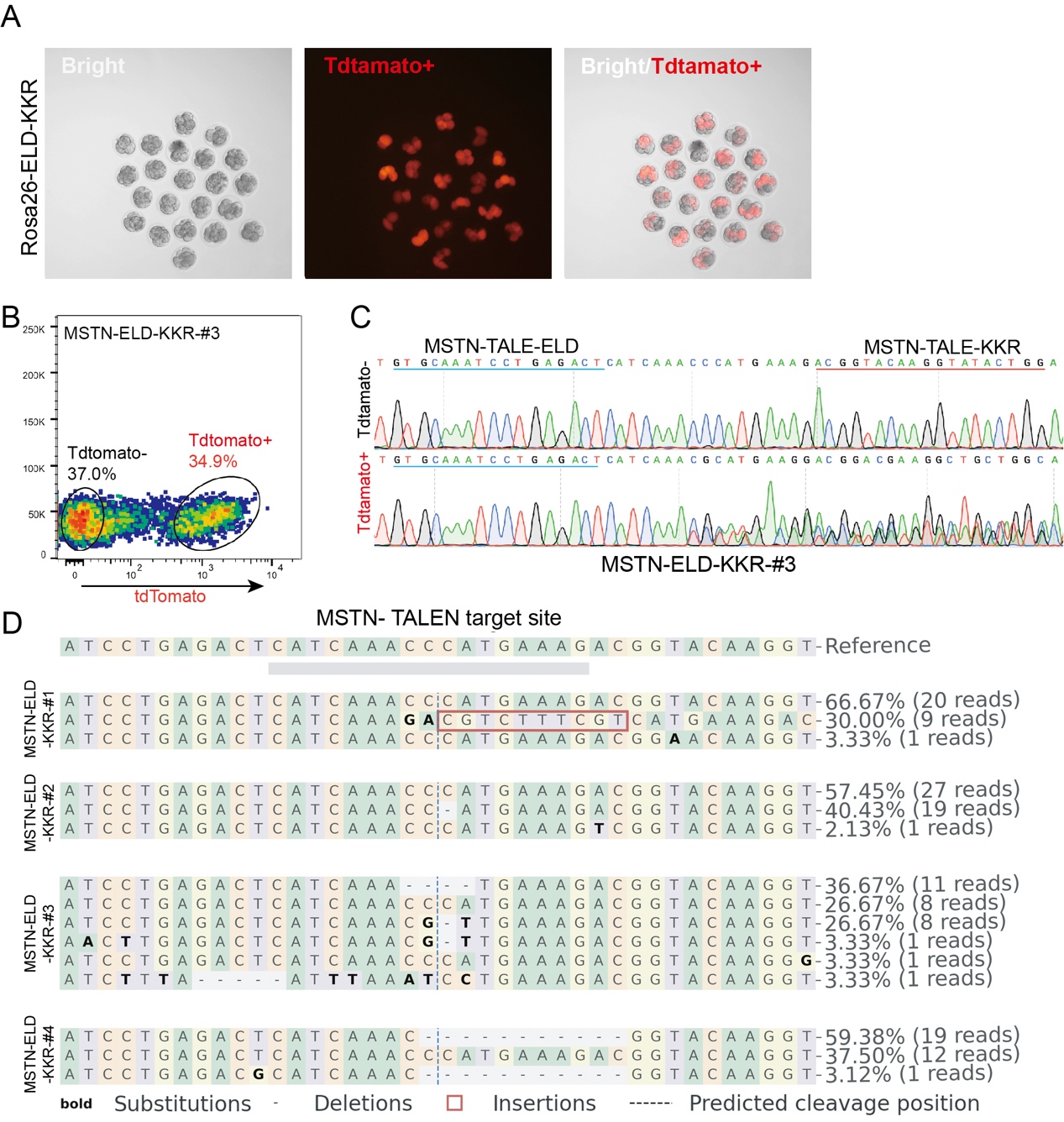
**

**Fig S6. Heterodimer-FokI-based TALENs architectures mediated MSTN gene editing in one blastmere of 2-cell mouse embryos.**

**A.** The tdTomato expression of the 8-cells embryos which were edited one blastomere by MSTN-ELD-KKR + Cre at the two-cell stage observed by fluorescent microscope. **B.** FACS analysis of E14.5 embryos treated by MSTN-ELD-KKR + Cre. **C.** Sanger sequencing showed the on-target effects of tdTomato^+^ and tdTomato^-^ embryo cells treated by MSTN-TALE-ELD-KKR. **D.**WGS analysis showing editing effect of targeted MSTN-ELD-KKR in tdTomato^+^ embryo cells.

**
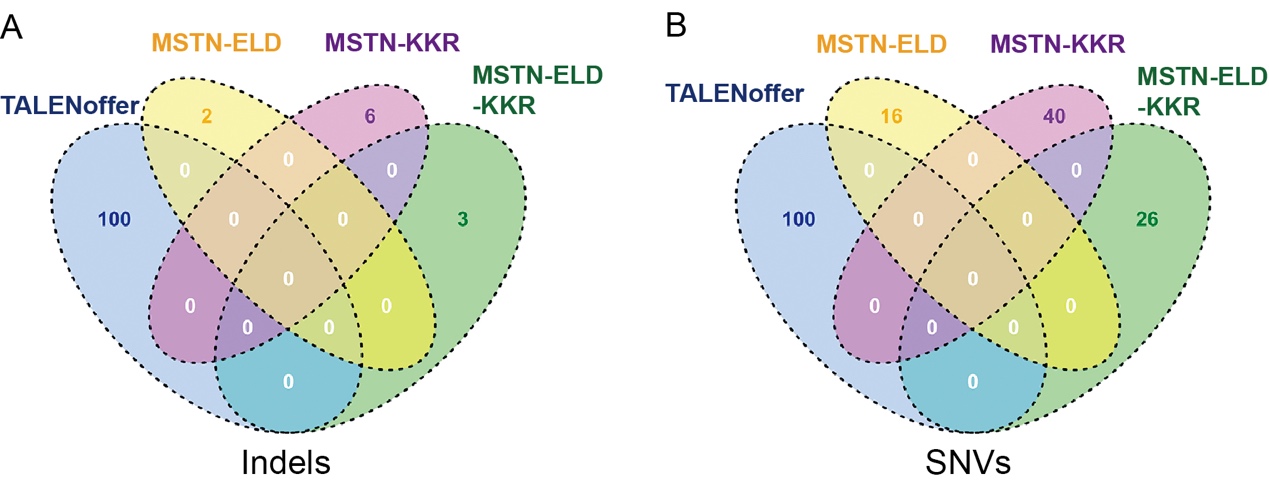
**

**Fig S7. Characteristic analysis showing unpredictable mutations in heterodimer-FokI-based TALENs architectures.**

**A, B.** Overlap among Indels (**A**) and SNVs (**B**) of MSTN-TALE-ELD, MSTN-TALE-KKR and MSTN-TALE-ELD-KKR detected by GOTI with predicted off-targets by TALENoffer;

**
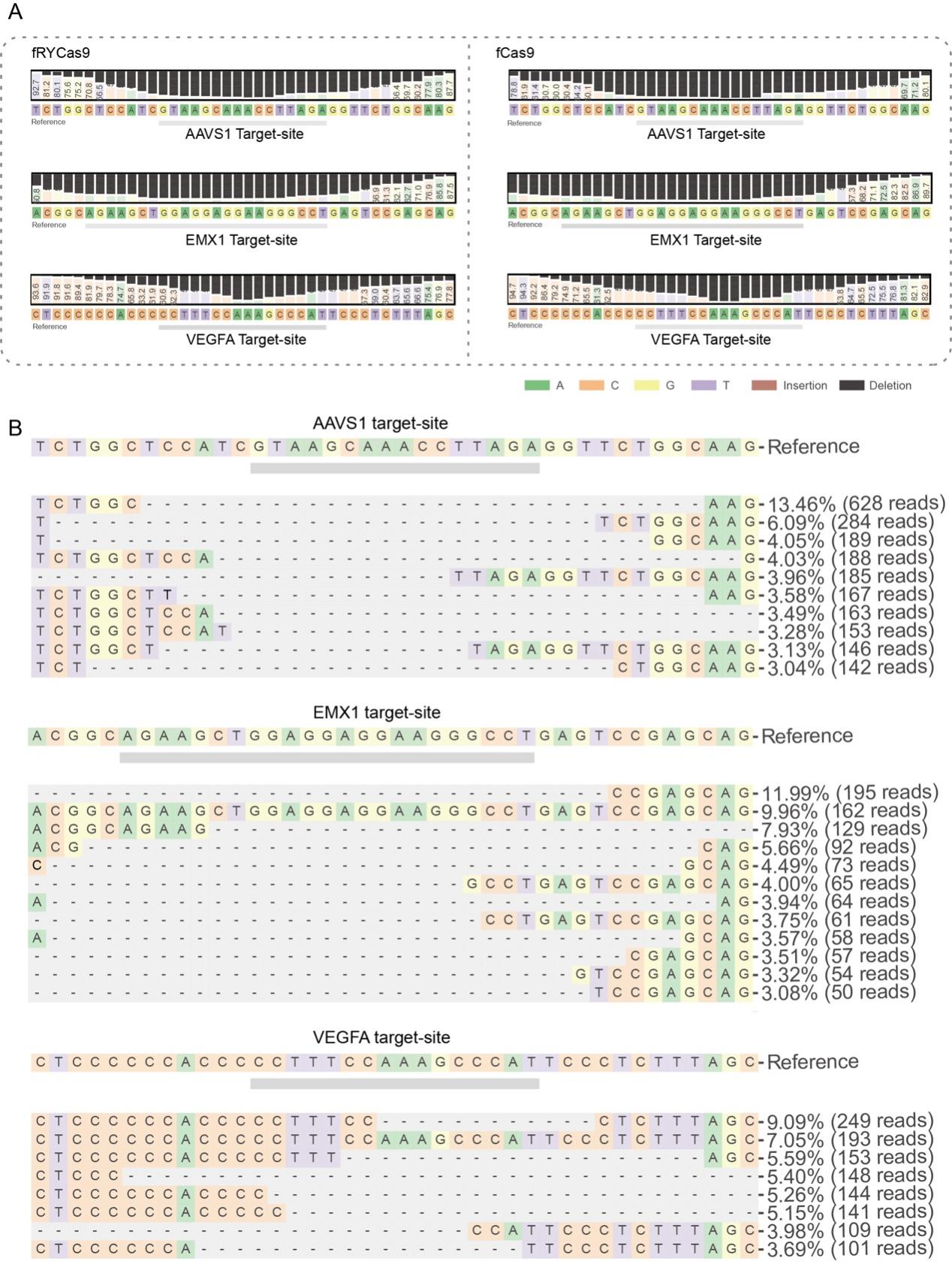
**

**Fig S8. fRYdCas9 meditated genome editing in HEK293T cells.**

**A.** Gene mutation nucleotide frequency in quantification window of fCas9 and fRYdCas9 in AAVS1, EMX1 and VEGFA loci. **B.** Gene mutation allele plots of fRYdCas9 in AAVS1, EMX1 and VEGFA loci.


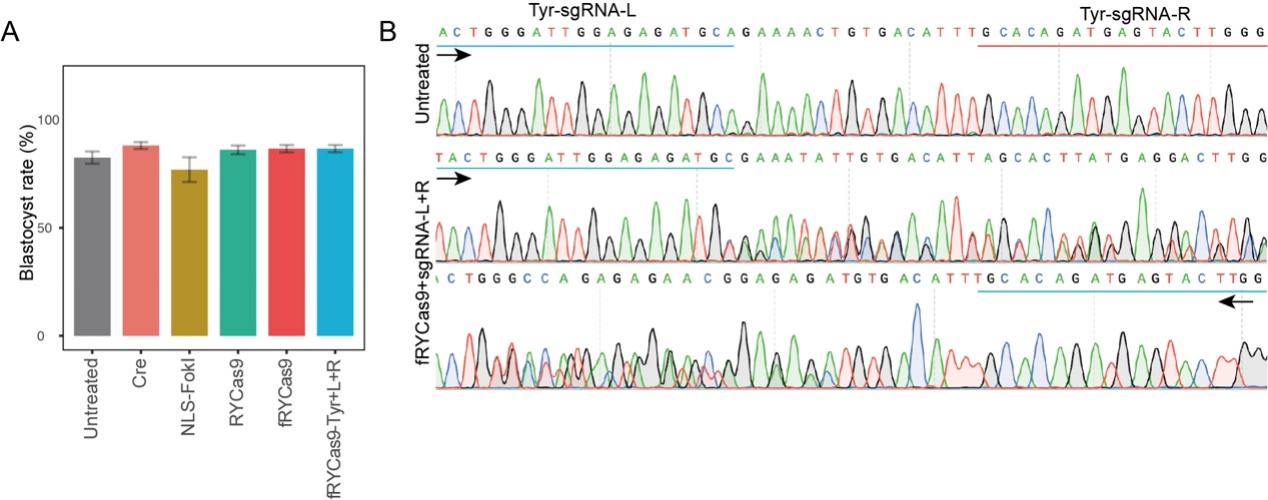


**Fig S9. Homodimer-FokI-based fRYdCas9 architectures mediated genome editing in mouse zygotes.**

**A.** Blastocyst rate of treated by Cre, FokI, RYCas9, fRYdCas9, or fRYdCas9-Tyr-L+R. **B.** Sanger sequencing analysis showing the gene editing effects of untreated and fRYdCas9-Tyr-L+R injected groups in E4.5 embryos (arrow represented Sanger sequencing direction). All *P* values were calculated by two-sided Student’s *t*-tests. n ≥ 3 replicates were used in all experiments.

**
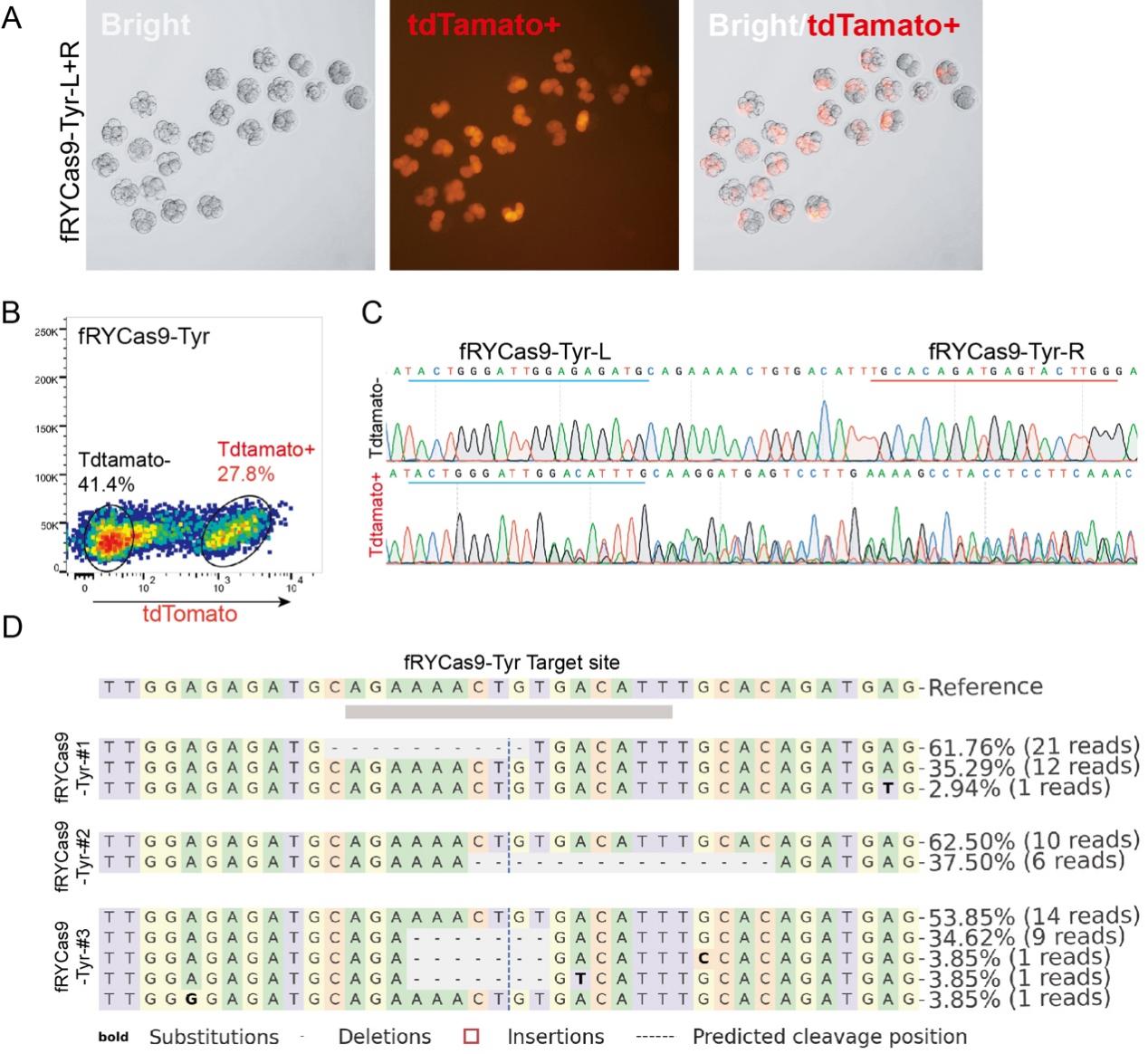
**

**Fig S10. Homodimer-FokI-based fRYdCas9 architectures mediated Tyr gene editing in one blastmere of 2-cell mouse embryos.**

**A.** The tdTomato expression of the 8-cells embryos which were edited one blastomere by fRYdCas9-Tyr-L+R + Cre at the two-cell stage observed by fluorescent microscope. **B.** FACS analysis of E14.5 embryos treated by fRYdCas9-Tyr-L+R + Cre. **C.** Sanger sequencing showed the on-target effects of tdTomato^+^ and tdTomato^-^ embryo cells treated by fRYdCas9-Tyr-L+R + Cre. **D.**WGS analysis showing editing effect of targeted fRYdCas9-Tyr-L+R + Cre in tdTomato^+^ embryo cells.


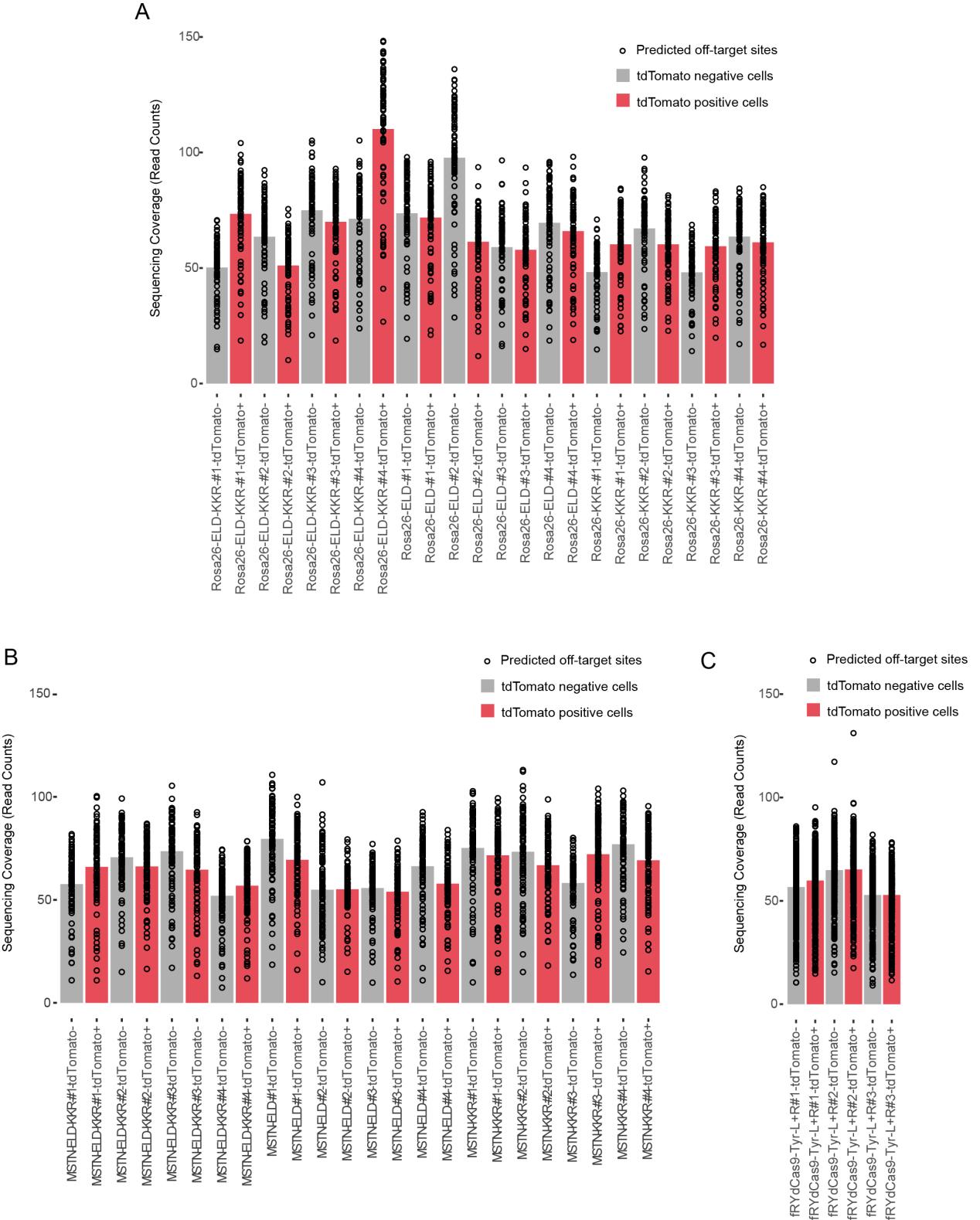


**Fig S11. The sequencing coverage of predicted off-target sites in GOTI samples.**

**A-C.** Predicted off-target sites sequencing coverage of targeted TALEN-Rosa26 (A), TALEN-MSTN (B), and fRYdCas9-Tyr (C) GOTI samples.

**Table S1. Summary of Indels and SNVs identified from WGS in each embryo.**

| **Group** | **on-target mutations** | **off-target Indels** | **Off-target SNVs** | **Exon off-target Indels** | **Exon off-target SNVs** | **Frameshift off-target**  **Indels** | **Nonsynonymous SNVs** |
| --- | --- | --- | --- | --- | --- | --- | --- |
| **Cre-#1** | 0 | 0 | 0 | 0 | 0 | 0 | 0 |
| **Cre-#2** | 0 | 0 | 0 | 0 | 0 | 0 | 0 |
| **Cre-#3** | 0 | 2 | 10 | 0 | 1 | 2 | 9 |
| **Rosa26-ELD-KKR-#1** | 1 | 1 | 6 | 0 | 0 | 1 | 6 |
| **Rosa26-ELD-KKR-#2** | 1 | 2 | 8 | 0 | 0 | 2 | 8 |
| **Rosa26-ELD-KKR-#3** | 1 | 1 | 11 | 0 | 1 | 1 | 10 |
| **Rosa26-ELD-KKR-#4** | 1 | 0 | 14 | 0 | 0 | 0 | 14 |
| **Rosa26-ELD-#1** | 0 | 0 | 2 | 0 | 0 | 0 | 2 |
| **Rosa26-ELD-#2** | 0 | 0 | 4 | 0 | 0 | 0 | 4 |
| **Rosa26-ELD-#3** | 0 | 0 | 2 | 0 | 0 | 0 | 2 |
| **Rosa26-ELD-#4** | 0 | 2 | 1 | 0 | 0 | 2 | 1 |
| **Rosa26-ELD-#5** | 0 | 0 | 2 | 0 | 0 | 0 | 2 |
| **Rosa26-KKR-#1** | 0 | 0 | 3 | 0 | 0 | 0 | 3 |
| **Rosa26-KKR-#2** | 0 | 0 | 10 | 0 | 0 | 0 | 10 |
| **Rosa26-KKR-#3** | 0 | 1 | 8 | 0 | 0 | 1 | 8 |
| **Rosa26-KKR-#4** | 0 | 1 | 11 | 0 | 0 | 1 | 11 |
| **MSTN-ELD-KKR-#1** | 1 | 0 | 2 | 0 | 1 | 0 | 2 |
| **MSTN-ELD-KKR-#2** | 1 | 1 | 10 | 1 | 0 | 0 | 10 |
| **MSTN-ELD-KKR-#3** | 1 | 1 | 2 | 0 | 0 | 1 | 2 |
| **MSTN-ELD-KKR-#4** | 1 | 3 | 12 | 1 | 1 | 2 | 11 |
| **MSTN-ELD-#1** | 0 | 0 | 1 | 0 | 0 | 0 | 1 |
| **MSTN-ELD-#2** | 0 | 0 | 6 | 0 | 0 | 0 | 6 |
| **MSTN-ELD-#3** | 0 | 1 | 4 | 0 | 0 | 1 | 4 |
| **MSTN-ELD-#4** | 0 | 1 | 5 | 0 | 0 | 1 | 5 |
| **MSTN-KKR-#1** | 0 | 1 | 11 | 0 | 0 | 1 | 11 |
| **MSTN-KKR-#2** | 0 | 1 | 17 | 0 | 0 | 1 | 17 |
| **MSTN-KKR-#3** | 0 | 4 | 12 | 0 | 0 | 4 | 12 |
| **MSTN-KKR-#4** | 0 | 0 | 0 | 0 | 0 | 0 | 0 |
| **NLS-ELD+NLS-KKR-#1** | 0 | 0 | 4 | 0 | 0 | 0 | 4 |
| **NLS-ELD+NLS-KKR-#2** | 0 | 0 | 4 | 0 | 0 | 0 | 4 |
| **NLS-ELD+NLS-KKR-#3** | 0 | 0 | 0 | 0 | 0 | 0 | 0 |
| **NLS-ELD+NLS-KKR-#4** | 0 | 0 | 5 | 0 | 0 | 0 | 5 |
| **NLS-ELD+NLS-KKR-#5** | 0 | 1 | 3 | 0 | 0 | 1 | 3 |
| **NLS-ELD+NLS-KKR-#6** | 0 | 0 | 13 | 0 | 0 | 0 | 13 |
| **NLS-ELD+NLS-KKR-#7** | 0 | 0 | 5 | 0 | 0 | 0 | 5 |
| **NLS-ELD-FokI-#1** | 0 | 0 | 12 | 0 | 0 | 0 | 12 |
| **NLS-ELD-FokI-#2** | 0 | 1 | 16 | 0 | 0 | 1 | 16 |
| **NLS-ELD-FokI-#3** | 0 | 1 | 3 | 0 | 0 | 1 | 3 |
| **NLS-ELD-FokI-#4** | 0 | 1 | 5 | 0 | 0 | 1 | 5 |
| **NLS-KKR-FokI-#1** | 0 | 0 | 6 | 0 | 0 | 0 | 6 |
| **NLS-KKR-FokI-#2** | 0 | 0 | 0 | 0 | 0 | 0 | 0 |
| **NLS-KKR-FokI-#3** | 0 | 1 | 14 | 0 | 0 | 1 | 14 |
| **NLS-KKR-FokI-#4** | 0 | 2 | 4 | 0 | 0 | 2 | 4 |
| **FokI-#1** | 0 | 0 | 5 | 0 | 0 | 0 | 5 |
| **FokI-#2** | 0 | 1 | 3 | 0 | 0 | 1 | 3 |
| **FokI-#3** | 0 | 0 | 9 | 0 | 0 | 0 | 9 |
| **fRYdCas9-#1** | 0 | 1 | 8 | 0 | 0 | 1 | 8 |
| **fRYdCas9-#2** | 0 | 1 | 5 | 0 | 0 | 1 | 5 |
| **fRYdCas9-#3** | 0 | 2 | 5 | 0 | 0 | 2 | 5 |
| **fRYdCas9-Tyr-#1** | 1 | 1 | 5 | 1 | 0 | 0 | 5 |
| **fRYdCas9-Tyr-#2** | 1 | 4 | 12 | 1 | 0 | 3 | 12 |
| **fRYdCas9-Tyr-#3** | 1 | 2 | 12 | 1 | 0 | 1 | 12 |
| **fRYCas9-BE3-TyrL#-1** | 1 | 11 | 107 | 0 | 0 | 0 | 0 |
| **fRYCas9-BE3-TyrL#-2** | 1 | 4 | 62 | 1 | 2 | 1 | 1 |
| **fRYCas9-BE3-TyrL#-3** | 1 | 18 | 94 | 0 | 1 | 0 | 1 |
|  | | | | | | | |

*The Single TALENs architecture or Cas9/RYCas9/fCas9/fRYdCas9-only group has no on-target sites, therefore their on-target effcieincy count as 0.

**Table S2. Indels from different calling algorithms.**

| **Group** | **Scalpel** | **Mutect** | **Strekla** | **Scalpel vs Mutect** | **Scalpel vs Strekla** | **Mutect vs Strekla** | **Overlap of 3 mtethods** | |
| --- | --- | --- | --- | --- | --- | --- | --- | --- |
| **Cre-#1** | 865 | 44 | 3224 | 0 | 0 | 0 | 0 |  |
| **Cre-#2** | 1166 | 50 | 4743 | 0 | 0 | 0 | 0 |  |
| **Cre-#3** | 3157 | 80 | 6808 | 2 | 2 | 2 | 2 |  |
| **MSTN-ELD-KKR-#1** | 645 | 30 | 2610 | 0 | 0 | 0 | 0 |  |
| **MSTN-ELD-KKR-#2** | 822 | 47 | 2754 | 0 | 0 | 0 | 0 |  |
| **MSTN-ELD-KKR-#3** | 730 | 29 | 2489 | 1 | 1 | 1 | 1 |  |
| **MSTN-ELD-KKR-#4** | 779 | 55 | 2938 | 2 | 2 | 2 | 2 |  |
| **MSTN-ELD-#1** | 731 | 19 | 2473 | 0 | 0 | 0 | 0 |  |
| **MSTN-ELD-#2** | 756 | 45 | 2413 | 0 | 0 | 0 | 0 |  |
| **MSTN-ELD-#3** | 994 | 24 | 3468 | 1 | 1 | 1 | 1 |  |
| **MSTN-ELD-#4** | 906 | 44 | 3133 | 1 | 1 | 1 | 1 |  |
| **MSTN-KKR-#1** | 1056 | 28 | 3019 | 1 | 1 | 1 | 1 |  |
| **MSTN-KKR-#2** | 746 | 38 | 2375 | 1 | 1 | 1 | 1 |  |
| **MSTN-KKR-#3** | 679 | 37 | 2723 | 4 | 4 | 4 | 4 |  |
| **MSTN-KKR-#4** | 723 | 17 | 2611 | 0 | 0 | 0 | 0 |  |
| **Rosa26-ELD-KKR-#1** | 438 | 14 | 972 | 0 | 0 | 0 | 0 |  |
| **Rosa26-ELD-KKR-#2** | 352 | 19 | 1269 | 2 | 2 | 2 | 2 |  |
| **Rosa26-ELD-KKR-#3** | 233 | 18 | 972 | 0 | 0 | 0 | 0 |  |
| **Rosa26-ELD-KKR-#4** | 341 | 36 | 1679 | 0 | 0 | 0 | 0 |  |
| **Rosa26-ELD-#1** | 572 | 21 | 1836 | 0 | 0 | 0 | 0 |  |
| **Rosa26-ELD-#2** | 477 | 23 | 2061 | 0 | 0 | 0 | 0 |  |
| **Rosa26-ELD-#3** | 618 | 24 | 2048 | 0 | 0 | 0 | 0 |  |
| **Rosa26-ELD-#4** | 489 | 23 | 1941 | 2 | 2 | 2 | 2 |  |
| **Rosa26-ELD-#5** | 552 | 27 | 1802 | 0 | 0 | 0 | 0 |  |
| **Rosa26-KKR-#1** | 463 | 30 | 2012 | 0 | 0 | 0 | 0 |  |
| **Rosa26-KKR-#2** | 978 | 42 | 2764 | 0 | 0 | 0 | 0 |  |
| **Rosa26-KKR-#3** | 440 | 27 | 1818 | 1 | 1 | 1 | 1 |  |
| **Rosa26-KKR-#4** | 569 | 23 | 2029 | 1 | 1 | 1 | 1 |  |
| **NLS-ELD-NLS-KKR-#1** | 585 | 26 | 1958 | 0 | 0 | 0 | 0 |  |
| **NLS-ELD-NLS-KKR-#2** | 771 | 30 | 2738 | 0 | 0 | 0 | 0 |  |
| **NLS-ELD-NLS-KKR-#3** | 524 | 31 | 1783 | 0 | 0 | 0 | 0 |  |
| **NLS-ELD-NLS-KKR-#4** | 393 | 23 | 1945 | 0 | 0 | 0 | 0 |  |
| **NLS-ELD-NLS-KKR-#5** | 534 | 35 | 2017 | 1 | 1 | 1 | 1 |  |
| **NLS-ELD-NLS-KKR-#6** | 816 | 49 | 3342 | 0 | 0 | 0 | 0 |  |
| **NLS-ELD-NLS-KKR-#7** | 559 | 18 | 2082 | 0 | 0 | 0 | 0 |  |
| **NLS-ELD-FokI-#1** | 663 | 42 | 2243 | 0 | 0 | 0 | 0 |  |
| **NLS-ELD-FokI-#2** | 804 | 29 | 2784 | 1 | 1 | 1 | 1 |  |
| **NLS-ELD-FokI-#3** | 561 | 34 | 2065 | 1 | 1 | 1 | 1 |  |
| **NLS-ELD-FokI-#4** | 624 | 55 | 2691 | 1 | 1 | 1 | 1 |  |
| **NLS-KKR-FokI-#1** | 511 | 39 | 2636 | 0 | 0 | 0 | 0 |  |
| **NLS-KKR-FokI-#2** | 427 | 114 | 3432 | 0 | 0 | 0 | 0 |  |
| **NLS-KKR-FokI-#3** | 445 | 29 | 1123 | 1 | 1 | 1 | 1 |  |
| **NLS-KKR-FokI-#4** | 579 | 23 | 1172 | 3 | 3 | 3 | 3 |  |
| **FokI-#1** | 779 | 26 | 2501 | 0 | 0 | 0 | 0 |  |
| **FokI-#2** | 758 | 32 | 2389 | 1 | 1 | 1 | 1 |  |
| **FokI-#3** | 846 | 29 | 2863 | 0 | 0 | 0 | 0 |  |
| **fRYdCas9-#1** | 1594 | 50 | 6273 | 4 | 4 | 4 | 4 |  |
| **fRYdCas9-#2** | 1262 | 69 | 5673 | 2 | 2 | 2 | 2 |  |
| **fRYdCas9-#3** | 1594 | 50 | 6273 | 4 | 4 | 4 | 4 |  |
| **fRYdCas9-Tyr-#1** | 632 | 43 | 2428 | 0 | 0 | 0 | 0 |  |
| **fRYdCas9-Tyr-#2** | 693 | 33 | 1977 | 4 | 4 | 4 | 4 |  |
| **fRYdCas9-Tyr-#3** | 1454 | 62 | 4575 | 4 | 4 | 4 | 4 |  |
| **fRYCas9-BE3-TyrL#-1** | 2024 | 115 | 7803 | 37 | 1887 | 77 | 11 |  |
| **fRYCas9-BE3-TyrL#-2** | 1666 | 27 | 6769 | 23 | 1579 | 15 | 4 |  |
| **fRYCas9-BE3-TyrL#-3** | 2723 | 82 | 8314 | 61 | 2455 | 45 | 18 |  |
|  | | | | | | | |  |

**Table S3. SNVs from different calling algorithms.**

| **Group** | **lofreq** | **Mutect** | **Strekla** | **lofreq vs Mutect** | **lofreq vs Strekla** | **Mutect vs Strekla** | **Overlap of 3 mtethods** | |
| --- | --- | --- | --- | --- | --- | --- | --- | --- |
| **Cre-#1** | 523 | 371 | 7867 | 0 | 0 | 0 | 0 |  |
| **Cre-#2** | 45 | 197 | 3771 | 0 | 0 | 0 | 0 |  |
| **Cre-#3** | 40 | 249 | 11966 | 10 | 10 | 10 | 10 |  |
| **MSTN-ELD-KKR-#1** | 23 | 96 | 2051 | 2 | 2 | 2 | 2 |  |
| **MSTN-ELD-KKR-#2** | 41 | 95 | 1599 | 10 | 10 | 10 | 10 |  |
| **MSTN-ELD-KKR-#3** | 35 | 86 | 1762 | 2 | 2 | 2 | 2 |  |
| **MSTN-ELD-KKR-#4** | 34 | 136 | 1705 | 12 | 12 | 12 | 12 |  |
| **MSTN-ELD-#1** | 15 | 72 | 1553 | 1 | 1 | 1 | 1 |  |
| **MSTN-ELD-#2** | 92 | 305 | 6612 | 6 | 6 | 6 | 6 |  |
| **MSTN-ELD-#3** | 58 | 113 | 1915 | 4 | 4 | 4 | 4 |  |
| **MSTN-ELD-#4** | 30 | 97 | 1934 | 5 | 5 | 5 | 5 |  |
| **MSTN-KKR-#1** | 27 | 84 | 1775 | 11 | 11 | 11 | 11 |  |
| **MSTN-KKR-#2** | 53 | 137 | 2619 | 17 | 17 | 17 | 17 |  |
| **MSTN-KKR-#3** | 34 | 99 | 1725 | 12 | 12 | 12 | 12 |  |
| **MSTN-KKR-#4** | 21 | 66 | 1778 | 0 | 0 | 0 | 0 |  |
| **Rosa26-ELD-KKR-#1** | 40 | 65 | 1910 | 6 | 6 | 6 | 6 |  |
| **Rosa26-ELD-KKR-#2** | 51 | 63 | 1447 | 8 | 8 | 8 | 8 |  |
| **Rosa26-ELD-KKR-#3** | 41 | 67 | 1081 | 11 | 11 | 11 | 11 |  |
| **Rosa26-ELD-KKR-#4** | 42 | 86 | 1278 | 14 | 14 | 14 | 14 |  |
| **Rosa26-ELD-#1** | 47 | 63 | 1739 | 2 | 2 | 2 | 2 |  |
| **Rosa26-ELD-#2** | 20 | 92 | 1813 | 4 | 4 | 4 | 4 |  |
| **Rosa26-ELD-#3** | 27 | 75 | 1997 | 2 | 2 | 2 | 2 |  |
| **Rosa26-ELD-#4** | 28 | 107 | 1808 | 1 | 1 | 1 | 1 |  |
| **Rosa26-ELD-#5** | 25 | 71 | 1893 | 3 | 3 | 3 | 3 |  |
| **Rosa26-KKR-#1** | 43 | 57 | 1907 | 3 | 3 | 3 | 3 |  |
| **Rosa26-KKR-#2** | 97 | 131 | 6646 | 10 | 10 | 10 | 10 |  |
| **Rosa26-KKR-#3** | 60 | 62 | 1659 | 8 | 8 | 8 | 8 |  |
| **Rosa26-KKR-#4** | 68 | 71 | 2128 | 11 | 11 | 11 | 11 |  |
| **NLS-ELD-NLS-KKR-#1** | 27 | 65 | 2432 | 4 | 4 | 4 | 4 |  |
| **NLS-ELD-NLS-KKR-#2** | 37 | 122 | 4174 | 4 | 4 | 4 | 4 |  |
| **NLS-ELD-NLS-KKR-#3** | 42 | 63 | 2049 | 0 | 0 | 0 | 0 |  |
| **NLS-ELD-NLS-KKR-#4** | 30 | 65 | 1453 | 5 | 5 | 5 | 5 |  |
| **NLS-ELD-NLS-KKR-#5** | 22 | 76 | 1870 | 3 | 3 | 3 | 3 |  |
| **NLS-ELD-NLS-KKR-#6** | 37 | 147 | 2683 | 13 | 13 | 13 | 13 |  |
| **NLS-ELD-NLS-KKR-#7** | 32 | 76 | 2464 | 5 | 5 | 5 | 5 |  |
| **NLS-ELD-FokI-#1** | 34 | 91 | 1893 | 12 | 12 | 12 | 12 |  |
| **NLS-ELD-FokI-#2** | 41 | 133 | 1762 | 16 | 16 | 16 | 16 |  |
| **NLS-ELD-FokI-#3** | 19 | 255 | 2203 | 3 | 3 | 3 | 3 |  |
| **NLS-ELD-FokI-#4** | 24 | 218 | 1794 | 5 | 5 | 5 | 5 |  |
| **NLS-KKR-FokI-#1** | 133 | 130 | 2134 | 6 | 6 | 6 | 6 |  |
| **NLS-KKR-FokI-#2** | 172 | 281 | 3020 | 0 | 0 | 0 | 0 |  |
| **NLS-KKR-FokI-#3** | 64 | 57 | 1654 | 14 | 14 | 14 | 14 |  |
| **NLS-KKR-FokI-#4** | 98 | 68 | 1915 | 4 | 4 | 4 | 4 |  |
| **FokI-#1** | 25 | 84 | 2040 | 5 | 5 | 5 | 5 |  |
| **FokI-#2** | 28 | 103 | 2046 | 3 | 3 | 3 | 3 |  |
| **FokI-#3** | 55 | 128 | 1958 | 9 | 9 | 9 | 9 |  |
| **fRYdCas9-#1** | 18 | 133 | 3648 | 5 | 5 | 5 | 5 |  |
| **fRYdCas9-#2** | 7 | 149 | 3871 | 5 | 5 | 5 | 5 |  |
| **fRYdCas9-#3** | 18 | 133 | 3648 | 5 | 5 | 5 | 5 |  |
| **fRYdCas9-Tyr-#1** | 17 | 95 | 1861 | 5 | 5 | 5 | 5 |  |
| **fRYdCas9-Tyr-#2** | 48 | 112 | 3205 | 12 | 12 | 12 | 12 |  |
| **fRYdCas9-Tyr-#3** | 35 | 119 | 3160 | 12 | 12 | 12 | 12 |  |
| **fRYCas9-BE3-TyrL#-1** | 812 | 375 | 4111 | 108 | 803 | 313 | 107 |  |
| **fRYCas9-BE3-TyrL#-2** | 1541 | 163 | 5566 | 62 | 1538 | 119 | 62 |  |
| **fRYCas9-BE3-TyrL#-3** | 1016 | 320 | 19550 | 94 | 1014 | 267 | 94 |  |
|  | | | | | | | |  |

**Table S4. Primer list for Nested PCR**

| Gene | Nested  PCR primer | Sequence (5'-3') |
| --- | --- | --- |
| Rosa26 | OF | ACTCGGGTGAGCATGTCT |
|  | OR | GGAGTGTTGCAATACCTTTCTG |
|  | IF | TTCAATTCCCCTGCAGGACA |
|  | IR | TTCTCTGCTGCCTCCTGGCTTC |
| MSTN | OF | ACAGCTGATGTTAAGGAGTGAG |
|  | OR | GTAAAAGCCCAACTGTGGAT |
|  | IF | GTTATCACTTACCAGCCCATC |
|  | IR | CAAAGCTTTGATTTCAATGCCT |
| AAVS1 | OF | GCAGCACCAGGATCAGTG |
|  | OR | GGTTAATGTGGCTCTGGTTC |
|  | IF | AGAACCGGGCAGGTCACG |
|  | IR | ACCTCCTGTTAGGCAGATTC |
| EMX1 | OF | CATCAGGCTCTCAGCTCAG |
|  | OR | GCAGCAAGCAGCACTCTG |
|  | IF | CTCCTGAGTTTCTCATCTGTGCC |
|  | IR | CCATTGGCCTGCTTCGTG |
| VEGFA | OF | GAAGCATCCCTGGACACTTC |
|  | OR | ACGTCCTCACTCTCGAAGAC |
|  | IF | GTACATGAAGCAACTCCAGTCCC |
|  | IR | CAGCTTCCCTGTGGTGGC |
| Tyr | OF | GGTTTCTGCCTTGGCACAG |
|  | OR | TGCAACTCTCTCTATATAGTGC |
|  | IF | CTTGTTATTGTGGGAACAAGAA |
|  | IR | GGATGCTGGGCTGAGTAAG |

OF, outer forward primer; OR, outer reverse primer; IF, inter forward primer; IR, inter reverse primer
