# Supplementary material for: Undetectable off-target effects induced by FokI catalytic domain in mouse embryos
Source: Genome Biol. 2024 Feb 20;25:51. doi: 10.1186/s13059-024-03188-9 (PMC10877887; doi:10.1186/s13059-024-03188-9)
Supplement: Supplementary file 3 — Additional file 3. Review history. [file 13059_2024_3188_MOESM3_ESM.docx]

Review History

**First round of review**

**Reviewer 1**

**Are you able to assess all statistics in the manuscript, including the appropriateness of statistical tests used?** Yes, and I have assessed the statistics in my report.

**Comments to author:**

To determine genome-wide off-target effects of various genome editing tools is a still important issue. In this manuscript, Xie et al. used Genome-wide Off-target analysis by Two-cell embryo Injection (GOTI) to detect off-target effects of TALEN or dCas9-FokI (named fCas9) systems. It is surprising that TALENs with FokI-heterodimers (ELD/KKR), FokI-homodimers fused RYdCas9 (fRYCas9), or even FokI catalytic domains alone (ELD/KKR heterodimer-FokI, homodimer-FokI) all resulted in no significant off-target effects. Considering that the GOTI is one of the most precise methods for detecting unbiased DNA off-target effects, this result would be valuable for further therapeutic applications. Overall, the manuscript is well written and the topic would be interesting for broad readers. It might be potentially suitable for this journal but I would like to raise a few issues to strengthen this study.

1) Because all data showed significant off-target effects, it is necessary to provide positive experiments that is well known to have genome-wide off-target effects previously. For example, In Figure 1B, wild-type (wt) SpCas9 or wt RYCas9 showed high on-target editing efficiencies. What about conducting the GOTI assay with those tools?

1-2) I could not understand the data in Figure 2C, in which no on-target editing were occurred by RYCas9. Is it different from the wt RYCas9 in Figure 2B data?

2) It is interesting that in Figure B, when the editing activity of RyCas9-R or RyCas9-L was relative low but (fRYCas9-L+R) was not low, which might imply that editing activity of RyCas9-R/L was poor but binding affinity of it was better. The reason should be discussed.

(minor)

3) For broad readers, it would be better to describe more details in the main text. For example, in Figure 1A, tdTomato positive cells indicated the transfection of mRNA of genome editing tools with Cre, not the genome edited cells.

- It is necessary to briefly introduce the RYCas9. How was it constructed and what was the PAM of it?

- For clear understanding, I recommend to revise the term of FokI-based RydCas9 (fRYCas9) to fRYdCas9, or how about 'RYdCas9-FolkI'?

- The following paper should be cited with ref #4, [PMID: 24770325]

**Reviewer 2**

**Are you able to assess all statistics in the manuscript, including the appropriateness of statistical tests used?** Yes, and I have assessed the statistics in my report.

**Comments to author:**

In the submitted manuscript, the authors reported their study on the specificity of different FokI-based constructs in genome targeting. They leveraged on mouse embryos for their study (GOTI) since one blastomere of a 2-cell embryo can be microinjected with the other uninjected blastomere serving as a control. From their results, they concluded that the FokI nuclease domain alone, either wildtype or mutated versions, was unlikely to contribute to off-target effects and that the "DNA-binding architecture (DBA)", such as TALE or dCas9, was more likely to be the culprit. Overall, the study provides some useful knowledge to the genome engineering and gene therapy communities. Nevertheless, the authors can strengthen their work further by more rigorous characterization of the different FokI-based constructs.

Major comments:

1) The experimental setup is not very well explained. For example, was Cre used as an injection control? If so, why couldn't the authors simply co-inject a mCherry mRNA? This would allow researchers to use embryos from different mouse strains, instead of being restricted to those from Ai9 (CAG-LoxP-Stop-LoxP-tdTomato) mice.

2) Line 123: The authors wrote that "none of the Indels overlapped with the predicted off-target sites". Is there sufficient sequencing coverage at the predicted off-target sites? There's a difference between high sequencing coverage with no variant read (0% editing) and poor sequencing coverage (inconclusive). Similarly, the same concern applies to several other parts of the results section where the authors claim that there's no overlap between predicted and observed off-targets.

3) Please state the number of SNVs for the Cre-only control group instead of writing "not significantly different". I believe there are few replicates, so it's hard to achieve statistical significance.

4) GOTI might not be the most sensitive method as it can be challenging to capture all possible modification events with WGS. I suggest using another complementary method to confirm the key conclusions. There are many possible methods to compare GOTI with, such as GUIDE-seq, DISCOVER-seq or DISCOVER-seq+, CAST-seq etc.

5) Human and mouse genomes have different complexities. I suggest confirming the key conclusions in human cells as well.

Minor comments:

6) Please explain what RYCas9 is upon first mention in the main text. Also, the authors use a number of labels without explaining what they mean.

7) This phrase on lines 137-139 is odd: "... zygotes injected with the FokI groups ... showed no significant difference in survival rate between the FokI groups and Cre injected groups". Do the authors simply mean there's no significant difference between the FokI groups and Cre injected groups?

8) Please state p-value and associated statistical test whenever significance (or lack of) is mentioned in the main text.

9) Please indicate the number of replicates in the figure legends.

**Authors Response**

**Point-by-point responses to the reviewers’ comments:**

Responses to the reviewers

(The responses to the reviewers are highlighted in blue)

Reviewer #1:

To determine genome-wide off-target effects of various genome editing tools is a still important issue. In this manuscript, Xie et al. used Genome-wide Off-target analysis by Two-cell embryo Injection (GOTI) to detect off-target effects of TALEN or dCas9-FokI (named fCas9) systems. It is surprising that TALENs with FokI-heterodimers (ELD/KKR), FokI-homodimers fused RYdCas9 (fRYCas9), or even FokI catalytic domains alone (ELD/KKR heterodimer-FokI, homodimer-FokI) all resulted in no significant off-target effects. Considering that the GOTI is one of the most precise methods for detecting unbiased DNA off-target effects, this result would be valuable for further therapeutic applications. Overall, the manuscript is well written and the topic would be interesting for broad readers. It might be potentially suitable for this journal but I would like to raise a few issues to strengthen this study.

Response: We thank the Reviewer for the time and constructive critique, which we believe have helped to improve the strength of our conclusions and the overall quality of our study. We have carefully considered all of the suggestions and done our best to address each comment. Hopefully the reviewer finds our responses and the additional experiments satisfy their concerns and that our paper is now suitable for publication.

1) Because all data showed no significant off-target effects, it is necessary to provide positive experiments that are well known to have genome-wide off-target effects previously. For example, In Figure 1B, wild-type (wt) SpCas9 or wt RYCas9 showed high on-target editing efficiencies. What about conducting the GOTI assay with those tools?

Response: Thank you for this valuable suggestion. We agree that a more sensitive GOTI experiment would provide stronger evidence supporting the high on-target and low (i.e., undetectable) off-target effects we presented in the originally submitted manuscript. As recommended, we used RYCas9-BE3+Tyr-L as a positive control for the GOTI assay in this manuscript. We thus transcripted the Tyr-L sgRNA and mRNA of Cre and RYCas9-BE3 in vitro, and injected the sgRNA and mRNA of Cre and RYCas9-BE3 into one blastomere of two-cell stage embryos. In E14.5, tdTotamo+/- cells were sorted by FACS for WGS sequencing.

Sequencing data showed that editing with RYCas9-BE3+Tyr-L resulted in 87.6±23.16 off-target events (SNVs) . By contrast, fRYdCas9 targeting of the same region resulted in fewer off-target effects in both Indel and SNV calling analyses. These results have been added to Figure 2C-E (Fig. R1). We again thank the reviewer for this suggestion, which has helped strengthen our conclusions.

**Fig. R1. (corresponds to Figure 2 in manuscripts). Undetectable off-target effect indued by homodimer-FokI-based fRYdCas9 in mouse embryos.**

A.Schematic of homodimer fRYdCas9. B. On-target efficiency of Cas9, RYCas9, fCas9, and fRYdCas9 for genes including AAVS1, EMX1, and VEGFA in HEK293T cells. C. On-target efficiency of tdTomato+ and tdTomato- cell for the treated groups including Cre, Fokl-Homo, RYCas9, fRYdCas9, and fRYdCas9-Tyr in mouse embryos. D, E. Comparison of the total number of detected Indels (D) and SNVs (E) for all groups. F, G. Overlap among Indels (F) and SNVs (G) of fRYdCas9-Tyr-L+R detected by GOTI with predicted off-targets by Casoffinder. All P values were calculated by two-sided Student’s t-tests. n ≥ 3 replicates were used in all experiments.Two Cre samples were derived from Zuo et al [DOI: 10.1126/science.aav9973 (2019)].

1-2) I could not understand the data in Figure 2C, in which no on-target editing were occurred by RYCas9. Is it different from the wt RYCas9 in Figure 2B data?

Response: Thank you for pointing out this source of potential confusion in our original manuscript, which we now understand could be unintentionally misleading for readers. As far as we know, the editing properties of FokI-RYdCas9 (fRYdCas9), which is based on RYdCas9, have not been characterized until this article. We therefore compared the on-target efficiency of the fRYdCas9 tool with that of RYCas9 and FokI-dCas9 by amplicon sequencing in HEK293T cells (Figure 2B). However, in Figure 2C, we focused on the sgRNA-independent off-target effects of RYCas9, and thus no sgRNAs were included. We have also modified our description of these results in the main text to clarify this point:

“ To further investigate potential off-target effects of homodimer FokI in the genome, we used GOTI to assess the FokI, RYCas9-only, fRYCas9-only, and fRYCas9-Tyr-L+R groups. ” (line184-186; Fig. 2C-E)

2) It is interesting that in Figure B, when the editing activity of RyCas9-R or RyCas9-L was relative low but (fRYCas9-L+R) was not low, which might imply that editing activity of RyCas9-R/L was poor but binding affinity of it was better. The reason should be discussed.

Response: We concur with the reviewer’s perspective. We found that fRYdCas9 exhibits better editing efficiency than RYCas9 at the same targeting site, possibly due to the higher DNA binding affinity of fRYdCas9. We agree that this point will raise interest in fRYdCas9 among readers and added text addressing this topic in the revised manuscripts as:

“It is interesting to note that the editing activity of RYCas9+sgRNA-L and RYCas9+sgRNA-R was relatively low, while fRYdCas9+sgRNA-L+sgRNA-R did not exhibited higher activity in target sites hAAVS1, hEMX1, and hVEGFA . This suggests that the formation of FokI dimer possibly increased the DNA binding affinity of fRYdCas9 and presented higher gene editing efficiency. ” (line 171-175)

(minor)

3) For broad readers, it would be better to describe more details in the main text. For example, in Figure 1A, tdTomato positive cells indicated the transfection of mRNA of genome editing tools with Cre, not the genome edited cells.

Responds: Thank you for this suggestion. We agree that more experimental detail will improve the accessibility of our study for a broad swath of readers who may be unfamiliar with these editing tools. In particular, we have revised the description for Figure 1A (line 106-108) and carefully checked the whole manuscript to improve the clarity of other similar sentences.

“After injection, the presence of Cre protein will remove the "-Stop-" component, initiating the expression of tdTomato. Consequently, the detection of tdTomato+ cells will serve as a reliable indicator of successful the delivery of mRNA of genome editing tools and Cre (Fig. S5A, S6A).” (Line108-111)

- It is necessary to briefly introduce the RYCas9. How was it constructed and what was the PAM of it?

Responds: Thank you for pointing out this oversight. We have added a brief introduction of RYCas9 in the main text , as well as a description of the plasmid construction process in the method section.

“To overcome the constraint imposed by target site recognition of a protospacer-adjacent motif (PAM), we used a nearly PAM-less (NNN, NRN > NYN) dead RYCas912 (RYdCas9) in place of dCas9 to construct FokI-RYdCas9 (fRYdCas9) (Fig. 2A).” (Line 160-162)

“Briefly, to construct fRYdCas9, FokI was fused to the N-terminus of RYdCas9 via a 32-amino acid linker sequence. Subsequently, a mCherry fluorescent protein was fused to the C-terminus of RYdCas9 using a T2A linker.” (Line 335-337)

- For clear understanding, I recommend to revise the term of FokI-based RydCas9 (fRYCas9) to fRYdCas9, or how about 'RYdCas9-FolkI'?

Response: Thank you for this valuable suggestion. As suggested, we have changed the term “FokI-based RYdCas9 (fRYCas9)” to “FokI-based RYdCas9 (fRYdCas9)” because it more clearly indicates that this is a “dead fRYCas9” variant, which is an essential point for understanding its experimental purpose.

- The following paper should be cited with ref #4, [PMID: 24770325]

(Dimeric CRISPR RNA-guided FokI nucleases for highly specific genome editing)

Respond: Thank you for providing this citation. We agree that this is an important study relevant to the current article as a reference for fRYdCas9. It is now included among the references.

We again thank you for your help with improving our manuscript. Your time and efforts spent in reviewing our study and providing constructive feedback are greatly appreciated and we hope you agree that the process has increased the quality of our paper, as intended.

Reviewer #2:

In the submitted manuscript, the authors reported their study on the specificity of different FokI-based constructs in genome targeting. They leveraged on mouse embryos for their study (GOTI) since one blastomere of a 2-cell embryo can be microinjected with the other uninjected blastomere serving as a control. From their results, they concluded that the FokI nuclease domain alone, either wildtype or mutated versions, was unlikely to contribute to off-target effects and that the "DNA-binding architecture (DBA)", such as TALE or dCas9, was more likely to be the culprit. Overall, the study provides some useful knowledge to the genome engineering and gene therapy communities. Nevertheless, the authors can strengthen their work further by more rigorous characterization of the different FokI-based constructs.

Response: We sincerely thank the Reviewer for their careful reading and constructive critique that has helped to reinforce our conclusions and improved the overall quality of our report. In general, these challenging questions have given us much to consider in the current study that will also guide our future research. We trust you will agree that the review process has functioned as intended to eliminate unclear data presentation and ensure the reliability and robustness of conclusions, resulting in considerable improvement of our article.

Major comments:

1) The experimental setup is not very well explained. For example, was Cre used as an injection control? If so, why couldn't the authors simply co-inject a mCherry mRNA? This would allow researchers to use embryos from different mouse strains, instead of being restricted to those from Ai9 (CAG-LoxP-Stop-LoxP-tdTomato) mice.

Response: Thank you for pointing out this potential source of confusion in our original manuscripts. We have rephrased the sentence explaining the experimental design to improve clarity.

“After injection, the presence of Cre protein will remove the "-Stop-" component, initiating the expression of tdTomato. Consequently, the detection of tdTomato+ cells will serve as a reliable indicator of successful the delivery of mRNA of genome editing tools and Cre (Fig. S5A, S6A).” (Line108-111)

Indeed, the mutations introduced by FokI-based editing tools are difficult to detect, especially in heterogeneous samples in which rare off-target mutations within individual cells may be masked at the population level. We developed the GOTI approach (Genome-wide Off-target analysis by Two-cell embryo Injection) to identify the potential off-target effects of gene editing tools in mouse embryos in vivo [DOI: 10.1126/science.aav9973, DOI: 10.1038/s41596-020-0361-11, DOI: 10.1016/j.cell.2021.04.035]. This method has since been successfully applied to detect the remarkably high off-target effects of Apobec [DOI: 10.1038/s41592-020-0832-x], DdCBE [DOI: 10.1038/s41421-022-00391-5], and others [DOI: 10.1126/sciadv.aba1773] and has facilitated the development of other new precise gene editing tools .

To address your question, there are a few reasons why mCherry mRNA is not used for the GOTI protocol:

a. Firstly, mCherry mRNA expression persists for only 2-3 days after injection in mouse embryos, after which mCherry fluorescence disappears. In this stage, embryonic cells will only expand into thousands of cells, which is not sufficiently abundant for WGS.

b. By contrast, injection of Cre mRNA efficiently induces a STOP region loss in Ai9(CAG-LoxP-Stop-LoxP-tdTomato) mice, resulting in long-term expression of the tdTomato reporter in edited blastomeres, and is also expressed in the progeny cells expanded from the edited cells. As a result, we are able to easily harvest enough cells for Indel and SNV detection by WGS.

However, please note that we greatly appreciate this innovative idea of injecting mCherry mRNA as a label. We now plan to test mCherry mRNA injection as a possible label for single-cell sequencing in future studies.

2) Line 123: The authors wrote that "none of the Indels overlapped with the predicted off-target sites". Is there sufficient sequencing coverage at the predicted off-target sites? There's a difference between high sequencing coverage with no variant read (0% editing) and poor sequencing coverage (inconclusive). Similarly, the same concern applies to several other parts of the results section where the authors claim that there's no overlap between predicted and observed off-targets.

Response: Thank you for raising this question, since this is a valid concern. Indeed, sufficient sequencing coverage is essential for determining off-target effects through GOTI and similar experiments. We have therefore added details of the sequencing data as supporting information in supplementary figures (Fig. R2; also in Fig. S11).

**Fig. R2. (corresponds to Supplemental Figure 11 in manuscripts). The sequencing coverage of predicted off-target sites in GOTI samples.**

**A-C.** Predicted off-target sites sequencing coverage of targeted TALEN-Rosa26 (A), TALEN-MSTN (B), and fRYdCas9-Tyr (C) GOTI samples.

In the GOTI protocol [DOI: 10.1038/s41596-020-0361-11], sequencing depth is set to 55x, and thus 50 or more reads should map to every gene locus. This coverage is enough to detect any off-target effects since base editing (and off-target effects) introduced in an early embryonic stage will be copied into progeny cells derived from the injected blastomere, thus amplifying the signal of off-target effects.

However, we agree that confirming the sequencing coverage in detected and predicted off-target sites is essential for rigorous and convincing evidence. We have added a step confirming the sequencing coverage into the standard GOTI protocol of our lab and added a mention of its importance in the revised article (line 366-368). We again thank the reviewer for suggesting this verification step.

“In addition, the sequencing coverage at the detected and predicted off-target sites were re-confirmed by Integrative Genomics Viewer (IGV).” (line 366-368)

3) Please state the number of SNVs for the Cre-only control group instead of writing "not significantly different". I believe there are few replicates, so it's hard to achieve statistical significance.

Response: Thanks for this constructive suggestion. We now provided the number of SNVs detected in the Cre-only control group in the revised manuscript ("n ≥ 3 replicates were used in all experiments" , Line 275-276). In this study, n ≥ 3 replicates were used in all experiments, which were based on several previously published experiments that reported successfully detecting the off-target effect of gene editing tools [DOI: 10.1126/science.aav9973, DOI: 10.1038/s41596-020-0361-11, DOI: 10.1038/s41421-022-00391-5, DOI: 10.1126/sciadv.aba1773, DOI: 10.1016/j.cell.2021.04.035].

In off-target analysis, no statistically significant differences was found between cells injected with the gene editing tools and the Cre-only group. As mentioned above, in previous studies we found that fewer than 10 off-target editing events (SNVs and Indels) can be detected in cells injected with Cre mRNA mRN, which is comparable with background mutations that arise during mouse development. In Figure 1 and Figure 2, we found 0-20 off-target counts in the experimental groups, which were not significantly different from that of the Cre-only group. These generally low variant counts similar to those in the Cre-only group support the reliability of the statistical analyses.

4) GOTI might not be the most sensitive method as it can be challenging to capture all possible modification events with WGS. I suggest using another complementary method to confirm the key conclusions. There are many possible methods to compare GOTI with, such as GUIDE-Seq, DISCOVER-seq or DISCOVER-seq+, CAST-seq etc.

Response: We appreciate this valuable suggestion of confirmation by an alternative method, since all methods are accompanied by advantages and disadvantages. GOTI is one of the most precise methods for detecting unbiased DNA off-target effects, and can be used to evaluate the probability of off-target effects of gene editing tools at the single-cell level with extremely low background. Since off-target effects introduced in early embryos are retained in the genomes of progeny cells expanded from the injected blastomere and amplified to population scale, WGS can easily and verifiably detect rare off-target edits by Sanger sequencing [DOI:10.1126/science.aav9973, DOI: 10.1126/science.aax1827]).

Despite our confidence that GOTI is one of the most sensitive and reliable approaches, we followed the reviewer’s suggestion and conducted a GUIDE-Seq experiment in mice N2a cells. We performed the GUIDE-Seq experiment following the step in the literature [DOI: 10.1038/nbt.3117 ]. Briefly, we transfected the plasmids (fRYdCas9+Tyr-L, fRYdCas9+Tyr-R, fRYdCas9+Tyr-L+Tyr-R, TALEN-Rosa26-ELD, TALEN-Rosa26-KKR, and TALEN-Rosa26-ELD-KKR ) into N2a cells, collected cells four days after transfection, and extracted the genome DNA. Genome DNA was sheared with a Covaris S200 instrument to an average length of 500 bp, end-repaired, A-tailed and ligated to half-functional adapters, incorporating a an 8-nt random molecular index. Two rounds of nested anchored PCR, with primers complementary to the oligo tag, were used for target enrichment. After the Illumina sequencing, data were first demultiplexed using Illumina’s bcl2fastq software and then analyzed.

In the GUIDE-Seq analysis, despite two off-target sites in the TALEN-Rosa26-ELD+KKR group, no off-target targets was found in other groups (Fig. R3). This result suggests that FokI-based gene editing exhibited limited off-target effects in mouse N2a cells, which is consistent with the data in GOTI assay in mouse embryos.

Fig. R3. Off-target effects detected by GUIDE-Seq for fRYdCas9 and TALEN groups in mouse N2a cells.

5) Human and mouse genomes have different complexities. I suggest confirming the key conclusions in human cells as well.

Response: We appreciate this valuable comment. As recommended, we also performed GUIDE-Seq analysis for off-target detection in human HEK293T cells. The experiment groups included : fRYdCas9+hCLTA-L, fRYdCas9+hCLTA-R, fRYdCas9+hCLTA-L+R, RYCas9+hCLTA-L, RYCas9+hCLTA-R,

fRYdCas9+hVEGFA-L, fRYdCas9+hVEGFA-R, fRYdCas9+hVEGFA-L+R, RYCas9+hVEGFA-L, RYCas9+hVEGFA-R.

Fig. R4. Off-target effects detected by GUIDE-Seq for fRYdCas9 groups in human HEK293T cells.

We barely detected off-target sites in fRYdCas9+hCLTA-L, fRYdCas9+hCLTA-R,, fRYdCas9+hCLTA-L+R, RYCas9+hVEGFA-L, fRYdCas9+hVEGFA-R, and fRYdCas9-hVEGFA-L+R group, while the off-target effects were relatively high in RYCas9+hCLTA-sgL/R, RYCas9+hVEGFA-sgL/R groups (Fig. R4). These results indicate that fRYdCas9 groups showed less off-target effects compared with RYCas9 groups in both target sites hCLTA and hVEGFA. This result suggests that FokI based gene editing exhibited limited off-target effects in human HEK293T cells, which is consistent with the data in the GOTI assay in mouse embryos.

We again thank the reviewer for this suggestion that we extend the scope of the investigation to include human cells.

Minor comments:

6) Please explain what RYCas9 is upon first mention in the main text. Also, the authors use a number of labels without explaining what they mean.

Response: Thanks for raising this point. We have corrected this issue in the text to ensure that RYCas9 is defined upon first mention in the manuscript and added a description of its gene editing characteristics (Line 73-74 and Line 159-161). We also carefully checked the whole text to make sure that all labels were clearly defined.

“Here, we used GOTI to evaluate the off-target effects induced by heterodimeric FokI-based TALENs and homodimeric FokI-based RYdCas9 (fRYdCas9, derived from a nearly PAM-less RYdCas9 variant^12^), as well as for the FokI catalytic monomer. ” (Line 72-75)

“To overcome the constraint imposed by target site recognition of a protospacer-adjacent motif (PAM), we used a nearly PAM-less (NNN, NRN > NYN) dead RYCas9^12^ (RYdCas9) in place of dCas9 to construct FokI-RYdCas9 (fRYdCas9) (Fig. 2A).” (Line 160-162)

7) This phrase on lines 137-139 is odd: "... zygotes injected with the FokI groups ... showed no significant difference in survival rate between the FokI groups and Cre injected groups". Do the authors simply mean there's no significant difference between the FokI groups and Cre injected groups?

Response: Thanks for pointing out this grammatical issue. We have corrected the sentence to now state as:

“In addition, the survival rate between NLS-ELD, NLS-KKR, NLS-ELD+NLS-KKR, and Cre at E4.5 (Fig. S4A) showed no significant difference” (Line 145-146)

8) Please state p-value and associated statistical test whenever significance (or lack of) is mentioned in the main text.

Response: Thanks for pointing out this oversight. We have added p-values and mentioned the associated statistical tests in the figure legends and the method section in the revised manuscript.

9) Please indicate the number of replicates in the figure legends.

Response: We have added the number of replicates to the figure legends.

**Second round of review**

**Reviewer 2**

Overall, the authors have addressed my previous concerns satisfactorily with their additional experiments. I just have a few remaining minor comments:

The authors wrote: "It is interesting to note that the editing activity of RYCas9+sgRNA-L and RYCas9+sgRNA-R was relatively low, while fRYdCas9+sgRNA-L+sgRNA-R did not exhibited higher activity in target sites hAAVS1, hEMX1, and hVEGFA."

(a) Should it be "fRYdCas9+sgRNA-L+sgRNA-R exhibited higher activity"?

(b) The authors hypothesized that "the formation of FokI dimer possibly increased the DNA binding affinity ..." However, another possibility is that with two gRNAs, the Cas9 enzyme has twice the number of chances of editing the target locus. To address this alternative hypothesis, the authors can test Cas9-L+R and RYCas9-L+R (without FokI).

**Authors Response**

**Point-by-point responses to the reviewers’ comments:**

Response to the Reviewer #2

*Reviewer #2: Overall, the authors have addressed my previous concerns satisfactorily with their additional experiments. I just have a few remaining minor comments:*

*The authors wrote: "It is interesting to note that the editing activity of RYCas9+sgRNA-L and RYCas9+sgRNA-R was relatively low, while fRYdCas9+sgRNA-L+sgRNA-R did not exhibited higher activity in target sites hAAVS1, hEMX1, and hVEGFA."*

*(a) Should it be "fRYdCas9+sgRNA-L+sgRNA-R exhibited higher activity"?*

*(b) The authors hypothesized that "the formation of FokI dimer possibly increased the DNA binding affinity ..." However, another possibility is that with two gRNAs, the Cas9 enzyme has twice the number of chances of editing the target locus. To address this alternative hypothesis, the authors can test Cas9-L+R and RYCas9-L+R (without FokI).*

Response: We sincerely appreciate the correction and valuable suggestions of the reviewer.

(a) We agree with the reviewer. We changed the text to "fRYdCas9 + sgRNA-L + sgRNA-R exhibited higher activity” to clarify this point. A description of the result has been added to the revised Results section as follows:

**“It is interesting to note that the editing activity of RYCas9 + sgRNA-L and RYCas9 + sgRNA-R was relatively low, while fRYdCas9+sgRNA-L+sgRNA-R exhibited higher activity in target sites hAAVS1, hEMX1, and hVEGFA.”**

(b) We sincerely appreciate the reviewer’s expert analysis. To test the hypothesis, we performed and incorporated the relevant experiments for Cas9-L+R and RYCas9-L+R (without FokI). The data from these experiments support the hypothesis that the addition of Cas9-L+R or RYCas9-L+R enhances editing efficiency, the description of these findings has been added to the revised Results section as follows:

**“For SpCas9 and RYCas9, employing a double sgRNA strategy may enhance the likelihood of editing the target locus, resulting in a higher percentage of modifications observed in the gene locus AAVS1 (SpCas9:93.867 ± 1.743 %; RYCas9:80.425 ± 2.8714% ), EMX1 (SpCas9:76.759 ± 0.409 %; RYCas9:70.037 ± 12.376% ), and VEGFA (SpCas9:87.571 ± 0.455%; RYCas9:56.202 ± 3.722% ) (Fig. 2B). However, gene editing with fRYdCas9 only occurs when both sgRNAs bind to the target locus. This suggests that the formation of FokI dimer possibly increased the DNA binding affinity of fRYdCas9 and presented higher gene editing efficiency. ”**

Again, we deeply appreciate the reviewers for their time and highly constructive comments, which have helped us to substantially improve the clarity and quality of our study.
